# Molecular and genetic bases of heat stress responses in crop plants and breeding for increased resilience and productivity

**DOI:** 10.1093/jxb/eraa034

**Published:** 2020-01-23

**Authors:** Michela Janni, Mariolina Gullì, Elena Maestri, Marta Marmiroli, Babu Valliyodan, Henry T Nguyen, Nelson Marmiroli

**Affiliations:** 1 Institute of Bioscience and Bioresources (IBBR), National Research Council (CNR), Via Amendola, Bari, Italy; 2 Institute of Materials for Electronics and Magnetism (IMEM), National Research Council (CNR), Parco Area delle Scienze, Parma, Italy; 3 Department of Chemistry, Life Sciences and Environmental Sustainability, University of Parma, Parco Area delle Scienze, Parma, Italy; 4 Division of Plant Sciences, University of Missouri, Columbia, MO, USA; 5 Lincoln University, Jefferson City, MO, USA; 6 CINSA Interuniversity Consortium for Environmental Sciences, Parma/Venice, Italy; 7 University of Birmingham, UK

**Keywords:** Breeding, climate change, cultivated plants, food crops, food security, global warming, heat stress, omics, phenomics

## Abstract

To ensure the food security of future generations and to address the challenge of the ‘no hunger zone’ proposed by the FAO (Food and Agriculture Organization), crop production must be doubled by 2050, but environmental stresses are counteracting this goal. Heat stress in particular is affecting agricultural crops more frequently and more severely. Since the discovery of the physiological, molecular, and genetic bases of heat stress responses, cultivated plants have become the subject of intense research on how they may avoid or tolerate heat stress by either using natural genetic variation or creating new variation with DNA technologies, mutational breeding, or genome editing. This review reports current understanding of the genetic and molecular bases of heat stress in crops together with recent approaches to creating heat-tolerant varieties. Research is close to a breakthrough of global relevance, breeding plants fitter to face the biggest challenge of our time.

## Teaser

This review provides a wide-ranging assessment of approaches to reduce the impact of heat stress on food crops.

## Introduction

Current analysis conducted by scientific communities including NASA’s Goddard Institute for Space Studies (GISS) indicates that the average global temperature on Earth has increased by ~0.8 °C since 1880. Two-thirds of the warming has occurred since 1975, at a rate of roughly 0.15–0.20 °C per decade (Lorenz et al., 2019). Moreover, the Intergovernmental Panel on Climate Change (IPCC) in its last report assesses that even limiting global warming to just 1.5 °C would require an unprecedented change in many aspects of society (https://www.ipcc.ch/sr15/).

For many years, scientists have studied the physiological mechanisms underlying heat stress response (HSR) and tolerance in plants (Fahad et al., 2017; Prasad et al., 2017; Govindaraj et al., 2018). However, after the discovery of the molecular responses and regulation of heat shock proteins (HSPs) and the activation of other genes, understanding of the HSR process became more mechanistic (Key et al., 1981; Altschuler and Mascarenhas, 1982). Major advances have been made in the molecular mechanisms of HSR (Ohama et al., 2017) and identification of genes or quantitative trait loci (QTLs) associated with heat tolerance in plants.

The production of plants with new alleles in HS-responsive genes and the possibility of testing these plants on innovative phenotyping platforms offer the opportunity fully to establish the role of these genes in the HSR and to develop crop plants with an increased tolerance to heat (Comastri et al., 2018).

## Temperature stress, crop yield, and food security

Temperature is one of the major environmental factors which affect plant growth, development, and yield. Temperatures persistently above optimal for plant growth may induce HS and reduce yields. At some threshold, they will be lethal. Extreme heat events can be measured in different ways: the maximum temperatures reached (intensity), how often the events occur (frequency), and how long they last (duration). HS has negative effects on crop physiology (e.g. decreased photosynthesis, increased respiration) and plant growth and yield (Prasad et al., 2017). Another adverse effect of HS is the negative influence on the plant root system, which provides support, nutrient and water uptake, and transport to other plant organs (Valdés-López et al., 2016), resulting in disrupted pollination, flowering, root development, and root growth stages (Sehgal et al., 2017; Cho, 2018).

In many species, for both cool and warm seasons, yield reduction after HS is observed as a consequence of decreasing seed- or fruit-set percentage (Otero et al., 2011; Shah et al., 2011; Singh and Jwa, 2013; Jangid and Dwivedi, 2016; Fahad et al., 2017; Sehgal et al., 2017; Comastri et al., 2018; Devasirvatham and Tan, 2018; Govindaraj et al., 2018). In wheat, exposure to short episodes (2–5 d) of HS (>24 °C) at the reproductive stage (start of heading) resulted in substantial damage to floret fertility, while a mean daily temperature of 35 °C caused total failure. Increasing the duration of high temperature at this stage reduced the grain weight linearly (Maestri et al., 2002; Prasad and Djanaguiraman, 2014); similarly for pea (Bhattacharya, 2019), lentil (Barghi et al., 2012), and chickpea (Wang et al., 2006). An extensive review on the threshold temperatures for several crop species was reported by Kaushal et al., 2016. Table 1 shows threshold temperatures in vegetative and reproductive development for several crop species, together with literature indications about the losses (or gains) attributed to HS alone.

**Table 1.** Yield losses due to heat stress in cool and warm season crops

Species	Threshold temperatures for the species^*a*^	World production in 2017^*b*^ (kg ha^–1^)	Average yield reduction (%)	Reference
**Cool-season crops**				
Barley (*Hordeum vulgare* L.)	Not reported	3136	15	Weichert et al. (2017)
Chick pea (*Cicer arietinum* L.)	15–30 °C for growth, 25 °C for reproductive growth	1015	19–50	Devasirvatham and Tan (2018)
Citrus (*Citrus* spp.)	35 °C for vegetative growth	9600	N/A	N/A
Lentils (*Lens culinaris* Medik.)	Not reported	1153	38–58	Sita et al. (2018)
Spinach (*Spinacia oleracea* L.)	Not reported	29 993	50	Yan et al. (2016)
Wheat (*Triticum* spp.)	20–30 °C for vegetative growth, 15 °C for reproductive growth	3531	6	Lobell et al. (2011); Zampieri et al. (2017); Comastri et al. (2018)
**Warm-season crops**				
Grapes (*Vitis vinifera* L.)	Not reported	10 716	35–50	Greer and Weedon (2013)
Maize (*Zea mays* L.)	33–38 °C for photosynthesis and pollen viability	5755	7–40	Valdés-López et al. (2016); Zhao et al. (2017); Meseka et al. (2018); Prasad et al. (2018)
Peanut (*Arachis hypogaea* L.)	29–33 °C for vegetative growth, 39–40 °C for seed set and yield	1686	6	Prasad et al. (2001)
Potato (*Solanum tuberosum* L.)	Not reported	20 111	18–23	Hancock et al. (2014)
Rapeseed (*Brassica napus* L.)	About 30 °C for flowering	2195	Up to 85%	Koscielny et al. (2018); Sparks (2018)
Rice (*Oryza sativa* L.)	33 °C for biomass, 35 °C limiting for grain formation and yield	4602	3	Zhao et al. (2017)
Sorghum [*Sorghum bicolor* (L.) Moench]	26–34 °C for vegetative growth, 40 °C for reproductive growth and yield	1416	17–44	Tack et al. (2017)
Soybean [*Glycine max* (L.) Merr.]	26–36 °C for reproductive development, 39 °C lethal	2854	3–7	Valdés-López et al., 2016; Zhao et al. (2017)
Sunflower (*Helianthus annuus* L.)	Not reported	1804	10–70	Debaeke *et al.* (2017)
Tomato (*Solanum lycopersicum* L.)	37 °C for vegetative growth, 28–30 °C for reproductive development	37 600	28	Snider et al. (2012); Lamaoui et al. (2018)

^*a*^ Data from Luo (2011) and Kaushal *et al.* (2016).

*b* Data obtained by world production and world cultivated extension for each crop, from FAOSTAT 2017, http://www.fao.org/faostat/en/#data/QC/visualize.

HS can impair several physiological processes linked to seed size and quality. HS during grain filling markedly decreased starch accumulation in wheat (Hurkman et al., 2003), rice (Yamakawa and Hakata, 2010), and maize (Yang et al., 2018). The levels of sugars such as fructose, sugar nucleotides, and hexose phosphate also declined due to HS (Yang et al., 2018). The decrease in sugars may be related to assimilate utilization for purposes other than edible component production (Asthir et al., 2012). In maize, waxy grain starch content decreased, whereas protein content increased, resulting in a change of grain quality (Yang et al., 2018). Moreover, increasing temperature and CO_2_ reduced protein and micronutrient content in grain (Chakraborty and Newton, 2011) and soybean (Li et al., 2018). In soybean under HS, the nutritional value of total free amino acids was reduced together with total protein concentration, while the oil concentration was significantly increased. As a general evaluation, reductions in total yield are mainly due to an alteration in balance of the source and sink activities that take place under HS.

The capacity of crop plants to overcome temperature stress has been interpreted in terms of avoidance, escape, or tolerance (Osmond et al., 1987). Avoidance is any mechanism that permits the plant to be or to become non-susceptible to the stressor effect, annulling in this way most of its deleterious effects (Hasanuzzaman et al., 2013). In escape, plants can alter their growth cycle before the stress hits hard, such as anticipating reproduction or shedding vegetative structures. From the agronomic point of view, tolerance is certainly more interesting; being based on the endurance of the plant in stress conditions, it does not involve substantial modification of the growth habit with potential deleterious effects on yield. A heat-tolerant plant is therefore inclined to continue its growth cycle quite independently of the stress (Barnabás et al., 2008).

## The contributions of ‘omics’

Conventional plant breeding strategies based mainly on phenotype selection and qualitative genetics have led breeders, after the green revolution, to achieve continuous increases in seed yield and improved yield stability, independently of the environmental cost of achieving them (Setia and Setia, 2008). However, the growing worldwide demand for enhancing yields of major crops is placing pressure on breeding programs to provide elite cultivars more adaptable to the ongoing changes. The complexity of the information needed to meet this challenge has prompted advances in understanding the biochemical and molecular processes that underlie important metabolic, physiological, and developmental traits which affect the ability of plants to cope with the upcoming climate change. This has provided new insights into how plants function, and promoted the development of new scientific disciplines, mainly based on high-throughput approaches.

The progress of ‘omics’ technologies, in particular genomics, transcriptomics, proteomics, and metabolomics, has enabled direct and unbiased monitoring of the factors affecting crop growth and yield in response to environmental threats. Overall, omics constitute powerful tools to reveal the complex molecular mechanisms underlying plant growth and development, and their interactions with the environment, which ultimately determine yield, nutritional value (Setia and Setia, 2008; Soda and Wallace, 2015), and the required level of agricultural inputs. The following sections provide some examples showing the successful application of omics in crop improvement.

### Genomics and transcriptomics

Molecular responses to HS have been investigated in many plant species to identify the complex processes and pathways regulated in acclimation and protection against temperature stress (Qu et al., 2013; Driedonks *et al.*, 2016). Regulation of gene expression through transcriptional and post-transcriptional mechanisms is certainly linked to both stress recognition and stress response; this aspect has been investigated at different stress levels and comparing different genetic resources. The conventional transcriptomic approach has been applied to study gene expression changes under stress conditions in many crop species, such as rice (Sarkar et al., 2014; González-Schain et al., 2016; Mangrauthia et al., 2017), tomato (Frank et al., 2009; Bita et al., 2011), barley (Mangelsen et al., 2011), maize (Frey et al., 2015; Shi et al., 2017*b*), wheat (Qin et al., 2008), soybean (Chung et al., 2013; Gillman et al., 2019), brassica (Dong et al., 2015), and grape (Liu et al., 2012*a*). More recently, application of advanced genome-wide transcriptome analyses has elucidated the roles of relevant genes from HS perception to signal transduction and stress response (Table 2). In general, most of the HS-responsive genes are involved in primary and secondary metabolism, translation, transcription, regulation, and responses to processes such as calcium, phytohormone, sugar, and lipid signaling, or protein modifications including phosphorylation (Jha et al., 2014; Takahashi and Shinozaki, 2019). Up-regulation under HS conditions is confined mainly to transcription factors and HSPs.

**Table 2.** Review of the transcriptomic, proteomic and metabolomic analyses performed to dissect the mechanisms involved in the heat stress response

Species	Primary stress	Secondary stresses	Tissue or development stage analyzed	Simulation conditions	Molecular techniques applied	References
Barley	Heat	N/A	Anthesis	42 °C	Transcriptomics (Affymetrix Barley1 GeneChips)	Mangelsen et al. (2011)
Barley	Heat	Drought	Plant leaf	26 °C	Metabolomics IC-MS/MS	Templer et al., (2017)
Barley	Heat	Mild drought and combination	Flag leaves	30 °C+50% FC	Transcriptomics (RNA-seq)	Cantalapiedra et al. (2017)
Chickpea	Heat	N/A	Whole plant development	Up to 42 °C	Proteomics (LC-MS)	Parankusam et al. (2017)
Chickpea	Heat	General oxidative stress	Plant leaf	37 °C	Targeted metabolomics	Salvi et al. (2018)
Citrus	Heat	Drought	Plant leaf	40 °C	Metabolomics GC-MS LC-MS	Zandalinas et al. (2017)
Grape	Heat	Various temperatures	Leaves	35, 40, and 45 °C	Proteomics iTRAQ LC-MS/MS	Jiang et al. (2017)
Grape	Heat	Followed by recovery	Plant leaves	43 °C	Proteomics (iTRAQ LC-MS/MS)	Liu et al. (2014)
Grapes	Heat	N/A	Leaves	45 °C	Transcriptomics (Affymetrix gene chip)	Liu et al. (2012*a*)
Lentils	Heat	N/A	Pods	Up to 33 °C	Enzyme activities	Sita et al. (2018)
Lentils	Heat	N/A	Leaves and pods	30–50 °C	Enzyme activities	Chakraborty and Pradhan (2011)
Lentils	Heat	Drought, also combined	Leaves and pods	30 °C	Enzyme activities	Sehgal et al. (2017)
Maize	Heat	Drought	Leaves	42 °C	Proteomics (iTRAQ LC-MS/MS)	Hu et al. (2015); Zhao et al. (2016)
Maize	Heat	Drought	Chloroplasts	42 °C	Proteomics (MALDI TOF/ TOF MS/MS)	Hu et al. (2015)
Maize	Heat	N/A	Seedlings	32–38 °C	Transcriptomics (RNA-seq)	Frey et al. (2015)
Maize	Heat	N/A	Seedlings	42 °C	Transcriptomics (RNA-seq)	Shi et al. (2017*b*)
Maize	Heat	High CO_2_	Plant leaf	32 °C	Metabolomics (GC-MS)	Qu et al. (2018)
Mung bean	Heat	Exogenous glutathione	Plant leaves	42°	Targeted lipidomics	Nahar et al., 2015
Peanut	Heat	N/A	Seedlings	40 °C and 30 °C	Physiological measurements, metabolomics	Singh et al. (2016)
Peanut	Heat	N/A	Leaves	45 °C	Transcriptomics (qRT–PCR), physiological measurements	Chakraborty et al. (2018)
Potato	Heat	N/A	Leaves, tubers	Up to 30 °C	Metabolomic (GC/MS), physiological measurements, transcriptomic (microarray)	Hancock et al. (2014)
Potato	Heat, drought		Leaves	35 °C	Transcriptomics (RNAseq)	Tang et al. (2016)
Potato	Heat	*Potato virus Y* (PVY)	Leaves	28 °C	Transcriptomics (qRT–PCR)	Makarova et al. (2018)
Potato	Heat		Tubers, skin and phelloderm	33–35 °C	Transcriptomics (qRT–PCR)	Ginzberg et al. (2009)
Rapeseed	Heat	N/A	Flowering	Up to 35 °C	GWAs	Rahaman et al. (2018)
Rapeseed	Heat	N/A	Developing seed	Up to 35 °C	Transcriptomics (95k EST microarray)	Yu et al. (2014)
Rapeseed	Heat, drought	Combination of the two stresses		29±0.5 °C from the 38th day after sowing, 30% field capacity	Metabolomics, physiological measurements	Elferjani and Soolanayakanahally (2018)
Rice	Heat	N/A	Anthers	Up to 37 °C in	Proteomics (iTRAQ LC-MS/MS)	Mu et al. (2017)
Rice	Heat	N/A	Grain and Leaves	30 °C	Transcriptomics (qRT–PCR)	Phan et al. (2013)
Rice	Heat	Open field	Leaves, early milky stage of rice grains, spikelets	42/32, 38, 28 °C	Proteomics (MALDI TOF/TOF MS/MS)	Liao et al. (2014); Das et al. (2015)
Rice	Heat	Cold	Suspension cell	44 °C	Proteomics (label-free in-gel digestion together with LC-MS/MS)	Gammulla et al. (2010)
Rice	Heat	Various temperatures	Seedlings, anthesis	35 °C, 40 °C and 45 °C, 38 °C	Proteomics (MALDI TOF MS/MS)	Han et al. (2009); Jagadish et al. (2010)
Rice	Heat	N/A	Anthers	38 °C	Proteomics (label-free in-gel digestion together with LC-MS/MS)	Kim et al. (2015)
Rice	Heat	N/A	Plants at flowering	38 °C for 1 d	Transcriptomics (RNAseq, large-scale qRT–PCR)	González-Schain et al. (2016)
Rice	Heat	N/A	Seeds	Germination at 30 °C (control) and 42 °C (stress)	Transcriptomics (RNA-seq, qRT–PCR)	Mangrauthia et al. (2016)
Rice	Heat and drought	Sugar starvation	Floral organs		Metabolomic and Transcriptomics	Li et al. (2015)
Rice	Heat	Oxidative stress	10-day-old seedlings	42±1 °C for 30 min with 10 mM H_2_O_2_	Transcriptomics (60mer rice 44k array, qRT–PCR)	Mittal et al. (2012)
Rice	Heat	N/A	Pollen	37 °C	Targeted metabolomics	Feng et al. (2018)
Rice	Heat	N/A	Leaves	45 °C	Transcriptomics (RNA-seq)	Fang et al. (2018)
Rice	Heat	N/A	Grains	38 °C	Transcriptomics (RNA-seq)	Liao et al. (2015)
Rice	Heat	Drought exogenous IAA	Plant leaf	40 °C	Target lipidomics	Sharma et al. (2018)
Rice	Heat	Exogenous brassinosteroids	Plant leaf	47 °C	Target lipidomics	Thussagunpanit et al. (2015)
Sorghum	Heat	Drought	Seedlings	28 °C and 50 °C, water withholding. Combination of the stress	Transcriptomics (microarray)	Johnson et al. (2014)
Soybean	Heat	High humidity	Pre-harvest seed	40 °C	Proteomics (MALDI TOF/TOF MS/MS)	Wang et al. (2012)
Soybean	Heat	N/A	3- to 4-week-old plants	45 °C for 3, 6, 24 h	Transcriptomics (qRT–PCR)	Chung et al. (2013)
Soybean	Heat	High CO_2_	Plant leaf	36 °C	Metabolomics (GC-MS)	Xu et al. (2016)
Soybean	Heat	N/A	Roots	40 °C	Proteomics (LC-MS/MS); Transcriptomics (RNA-seq)	Valdés-López et al. (2016)
Soybean	Heat	N/A	Dry, imbibed, or germinated seeds from heat-tolerant and heat-sensitive cultivars	Field conditions with heat stress	Transcriptomics (RNA-seq)	Gillman et al. (2019)
Spinach	Heat	N/A	Leaves	37 °C, 35 °C	Physiological, enzyme activity, proteomic (trypsin digestion and iTRAQ labeling), transcriptomics (NGS)	Yan et al. (2016); Zhao *et al.* (2018)
Sunflower	Heat	Light	Leaves and immature seed	37 °C	Transcriptomics	Hewezi et al. (2008)
Tomato	Heat	Melatonin		Up to 42 °C	Metabolomics	Xu et al. (2016)
Tomato	Heat	Oxidative stress	Flower buds	36 °C\25 °C for 3 months	Proteomics (SDS–PAGE followed by LC-MS/MS)	Mazzeo et al. (2018)
Tomato	Heat	Lipid antioxidant and galactolipid remodeling	5- to 6-week-old plants	38 °C	Metabolomics (MS)	Spicher et al. (2016)
Tomato	Heat	N/A	Different developmental stages of tomato pollen	38 °C	Proteomics (mass accuracy precursor alignment (MAPA) plus LC/MS)	Chaturvedi et al. (2015)
Tomato	Heat	Waterlogging in open field	Leaves	39 °C	Proteomics (MALDI TOF/TOF MS/MS)	Lin et al. (2016)
Tomato	Heat	N/A	Anthers	32 °C for 2, 6, 16 or 30 h	Transcriptomics (cDNA-AFLP; 90 K Custom TomatoArray 1.0)	Bita et al. (2011)
Tomato	Heat	N/A	Flower buds	43–45 °C for 2 h	Transcriptomics (Affymetrix GeneChip® Tomato Genome Array)	Frank et al. (2009)
Tomato	Heat	N/A	week‐old tomato plants	1 h at 39 °C	Transcriptomics (massive analysis of cDNA ends (MACE)	Fragkostefanakis et al. (2015)
Tomato	Heat	N/A	Pollen	38 °C	Metabolomics (LC-QTOF-MS plus LTQ Orbitrap XL, mass spectrometer)	Paupière et al. (2017)
Tomato	Heat	N/A	Pollen tubes	37 °C	Targeted metabolomics	Muhlemann et al. (2018)
Tomato	Heat	Cold	Plant leaves	38, 20, 10 °C	Untargeted lipidomics LC/MS	Spicher et al. (2016)
Wheat	Heat	N/A	Seeds in filling stage	37 °C for 4 h	Metabolomics, transcriptomics	Wang et al. (2015)
Wheat	Heat	Lipid alteration	Flowering	Up to 35 °C	Metabolomics	Narayanan et al. (2016)
Wheat	Heat	N/A	Seedlings	Up to 42 °C	Transcriptomics	Comastri et al. (2018)
Wheat	Heat	N/A	Seedlings	35 °C	Proteomics (MALDI TOF/TOF MS/MS)	Gupta et al. (2015)
Wheat	Heat	N/A	Leaves, stems, and spikes, flag leaf	38 °C, 37 °C	Proteomics (iTRAQ LC-MS/MS)	Lu et al. (2017); Kumar et al. (2019)
Wheat	Heat	Open field	Grain	40 °C	Proteomics (MALDI TOF/TOF MS/MS)	Wang et al. (2018)
Wheat	Heat	Low nitrogen	Plant leaf	32 °C	Proteomics (MALDI TOF/TOF MS/MS)	Yousuf et al. (2017)
Wheat	Heat	Drought	Post-anthesis	35 °C	Proteomics (MALDI TOF/TOF MS/MS)	Zhang et al. (2018)
Wheat	Heat	N/A	Flag leaf	35 °C	Proteomics	Wang et al. (2015)
Wheat	Heat	N/A	Leaf of heat-susceptible ‘Chinese Spring’ (CS) and heat-tolerant ‘TAM107’ (TAM)	40 °C for 1 h with and without heat acclimation (34 °C, 3 h)	Transcriptomics (wheat genome array)	Qin et al. (2008)
Wheat	Heat	N/A	Flag leaf, seedlings	37/17 °C for 3 days in anthesis stage	Proteomics	Lu et al. (2017)
Wheat	Heat	N/A	Spikelet post-anthesis	35 °C	Metabolomics (LC/HRMS)	Thomason et al. (2018)
Wheat	Heat	Drought	Seedlings	40 °C, 20 % (w/v) PEG-6000	Transcriptomics (RNA-seq)	Liu et al. (2015)
Wheat	Heat	N/A	Plant leaf	35 °C	Lipidomics ICP-MS	Narayanan et al. (2016)
Wheat	Heat	N/A	Pollen	35 °C	Lipidomics ICP-MS	Narayanan et al. (2018)
Wheat	Heat	N/A	Plant leaf	35 °C	Lipidomics ICP-MS/MS	Djanaguiraman et al. (2018)

Abbreviations: cDNA-AFLP, cDNA-amplified fragment length polymorphism; GWAS, genome-wide association study; IAA, indole-3-acetic acid; IC-MS/MS, ion chromatography tandem MS; ICP-MS, inductively coupled plasma MS; iTRAQ, isobaric tags for relative and absolute quantitation; LC/HRMS, liquid chromatography-high resolution MS; MACE, massive analysis of cDNA ends; MALDI TOF/TOF MS/MS, matrix assisted laser desorption/ionization-time of flight tandem MS; MAPA, mass accuracy precursor alignment; N/A, not applicable; NGS, next-generation sequencing; qRT–PCR, quantitative reverse transcription–PCR; QTOF, quadrupole time of flight; RNA-seq, RNA sequencing,

The activation and production of heat shock factors (HSFs) and HSPs and the increase in reactive oxygen species (ROS)-scavenging activity play a key role in the responses and acclimation of plants to HS (Maestri et al., 2002; Driedonks et al., 2016; Comastri et al., 2018). ROS-related genes are within the main up-regulated category under HS in different plant species (Chao et al., 2008; Chou et al., 2012; Mittal et al., 2012; Suzuki et al., 2014) and have been associated with basal heat tolerance (Almeselmani et al., 2006, 2009; Kang et al., 2009; Bhattacharjee, 2012). Moreover, ROS play an important role as signal molecules involved in the transduction of intracellular and intercellular signals controlling gene expression and activity of anti-stress systems (Petrov and Van Breusegem, 2012), suggesting a role for ROS in the activation of HSFs during HS (Driedonks et al., 2016).

Both HSFs and HSPs are central to the HSR and in the acquisition of thermotolerance in plants (Scharf et al., 2012; Ohama et al., 2017). As a result of advances in whole-genome sequencing, additional information has been gathered for the *Hsp* gene family (Chen et al., 2018). HS transcription factors (TFs) involved in heat sensing and signaling are highly conserved both structurally and functionally throughout the eukaryotic kingdom and are key players for the expression of *Hsp* genes (Scharf et al., 2012). The *HSF* gene family has been identified and characterized in several crop species such as grape (Liu et al., 2012*b*), soybean (Scharf et al., 2012; Chung et al., 2013), maize (Lin et al., 2011), wheat (Xue et al., 2014), rice (Mittal et al., 2009; Jin et al., 2013; Lavania et al., 2018), and tomato (Yang et al., 2016). Progress in genome annotation has led to the development of the specialized HEATSTER database (http://www.cibiv.at/services/hsf/info#anfang) which can identify and correctly annotate new *HSF* genes in plant species (Berz et al., 2019).

The involvement of HSPs in the defense response following HS has been extensively reported in several plant species (Park and Seo, 2015; Comastri et al., 2018). Gene expression analysis was performed in tomato leaves applying MACE (massive analysis of cDNA ends), which showed that 2203 genes (9.6% of the total) had enhanced transcript abundance in response to HS (Fragkostefanakis et al., 2015). Those encoding small HSP (sHSP) family members showed the strongest induction, with nine out of 100 *Hsp40* genes up-regulated in response to heat. *HSF* genes showed basal and stable expression in non-stressed conditions and, for some, a strong induction in response to HS. These studies also reported the importance of TFs in the regulation of HSR, since 92 of the genes up-regulated upon HS are TF genes (*bHLH*, *bZIP*, *ERF*, *MYB*, and *WRKY* families). These are well-known major elements in HSR and thermotolerance, highlighting the complex regulatory networks activated beyond those directly controlled by HSFs. The tomato HsfA1a TF seems to have a unique function as a master regulator for acquired thermotolerance and cannot be replaced by any other HSF (Scharf et al., 2012).

In wheat, 6560 probe sets displayed changes in expression after heat treatment at 40 °C, with or without acclimation at 34 °C; representing *Hsp* genes, *HSF* genes, and genes encoding proteins involved in phytohormone biosynthesis/signaling, calcium and sugar signal pathways, RNA metabolism and ribosomes, as well as primary and secondary metabolism (Kotak et al., 2007; Qin et al., 2008). In barley, the effects of a short-term HS on central developmental functions of the caryopsis were studied at the transcriptional level (Mangelsen et al., 2011). Over 2000 differentially expressed genes were identified, equally divided between up- and down-regulation. The core HSR includes some conserved processes such as abscisic acid- (ABA) responsive gene activation, HSP-mediated protein folding, calcium signaling, ROS scavenging, and the biosynthesis of compatible solutes, which are rapidly induced. Within the down-regulated genes, it is noticeable that those involved in carbon and nitrogen metabolism and cellular growth, which determine long-term negative effects, differ significantly depending on the plant developmental stage at the time of the stress.

**Fig. 1.**
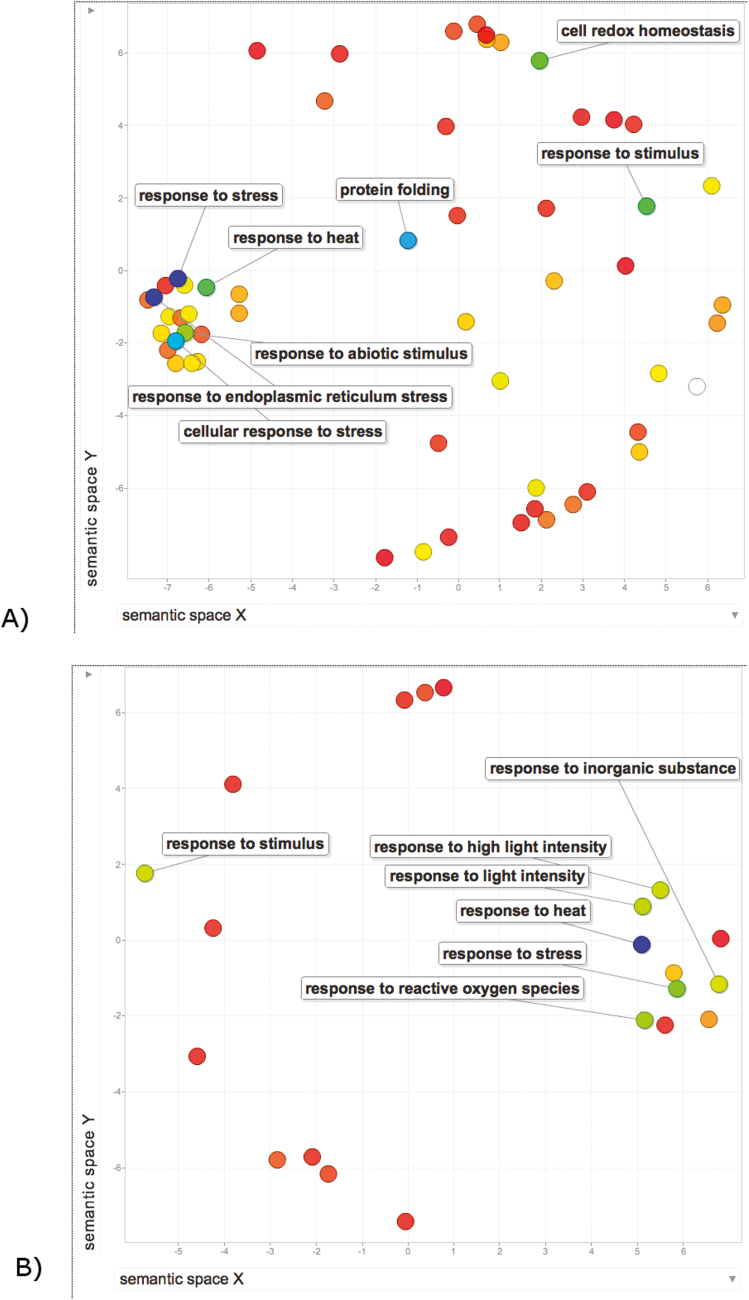
Scatterplots obtained by multidimensional scaling of the matrix of biological process Gene Ontology (GO) terms across transcriptomic data obtained from heat-stressed tomato (A) and maize (B). The color of each spot indicates the level of enrichment from red (lowest p) to blue (highest p). The labels refer only to the most frequent GO terms. (A) Tomato transcripts identified by Fragkostefanakis et al. (2015) are analyzed through their Arabidopsis orthologs, 67 genes in total; (B) maize transcripts have been identified in Frey et al. (2015) as significantly induced, 454 genes in total. Enriched GO classes have been identified with AgriGO v.2 (http://systemsbiology.cau.edu.cn/agriGOv2/;Du et al., 2010) and the results visualized with ReviGO (http://revigo.irb.hr;Supek et al., 2011).

During grain development, the main negative effects of HS are related to the decreased accumulation of storage compounds, which can have a detrimental effect on both seed quality and final yield (Mangelsen et al., 2011; Hurkman et al., 2013). In wheat, HSPs can play a crucial role in gluten formation as a ‘glue’ between the reserve proteins (glutenins and gliadins) and starch (Maestri et al., 2002). During flowering, a significant reduction in the expression of non-essential photosynthetic genes may constitute an energy-saving strategy to facilitate other key stress responses with the aim of maintaining sugar metabolism and consequently protecting pollen viability (X. Li et al., 2015). Unraveling the complexity of high temperature tolerance may benefit from a systems biology approach (see Fig. 1).

### Proteomics

Protein profiling under HS conditions helps to identify stress-responsive proteins, and the detailed investigation of these proteins reveals their function in stress tolerance mechanisms (Priya et al., 2019). The synthesis and accumulation of HSPs is a prompt response after exposure to high temperature and it is considered one of the most important adaptive strategies to overcome the deleterious effects of HS (Wahid et al., 2007; Keller and Simm, 2018). The majority of HSPs are molecular chaperones involved in protein stabilization and signal transduction during HS (Sung et al., 2001; Arce et al., 2018). HSPs prevent accumulation of proteins with anomalous conformations and eliminate non-native aggregations formed during stress, with ubiquitin-mediated degradation of these proteins (Kotak et al., 2007).

Proteomics allows the study of the direct gene products, which often differ with the level of gene regulation. This technique has been applied to investigate heat responses in rice, wheat, tomato, maize, soybean, grape, and chickpea (for references, see Table 2). The types of technique utilized for these studies have been mostly 2D gel electrophoresis (SDS–PAGE) for protein separation, followed by MS:MALDI-TOF (matrix-assisted laser desorption/ionization-time of flight), MS, or TOF/TOF MS/MS for protein isolation, and iTRAQ (isobaric tags for relative and absolute quantitation) with LC-MS/MS for relative quantification (Zhang et al., 2006; Wiese et al., 2007). Other techniques have been deployed including label-free in-gel digestion together with LC-MS/MS or Orbitrap; SDS–PAGE followed by LC-MS/MS; and mass accuracy precursor alignment (MAPA) plus LC-MS (Chaturvedi et al., 2015). Many successful examples of proteomics applied to the study of HSPs and other HS-induced proteins have been reported (Malcevschi and Marmiroli, 2012; Table 2).

Different stages of plant development have been investigated using proteomics, from seedling, to flower (anthers and pollen especially in rice and tomato), to fruit, but also spikelet and grain maturation. In rice, where HS has been studied in isolation, all classes of HSPs (high and low molecular weight) have been found among the predominant protein categories, although other protein types and Gene Ontology (GO) classes varied extensively according to the part of the plant investigated. For instance, in pollen and anthers, late embryogenesis abundant (LEA) proteins were present in high numbers. Interestingly, proteins related to oxidative stress were identified in most of the studies on high HS, alone or in combination with other stresses. ROS are produced during HSR, which results in an oxidative imbalance within the cell. Furthermore, chloroplast function and photosynthesis are both strongly affected by ROS generated during HS, and it seems that HSPs can prevent ROS damage to the photosystems or the organelle structure. As an example, in maize, it was found that sHSP26 protects chloroplasts under HS (Hu et al., 2015). Other GO categories involved were sugar metabolism, the tricarboxylic acid (TCA) cycle, regulatory proteins, and proteins that function in energy metabolism.

More information on stress-associated active proteins (SAAPs) in wheat has recently been reported (Kumar et al., 2019). This study identified ~4272 SAAPs in wheat using absolute quantification methods. Some of the differentially abundant SAAPs identified were Rubisco, Rubisco activase (RCA), oxygen-evolving extrinsic protein (OEEP), HSP17, superoxide dismutase (SOD), catalase (CAT), and calcium-dependent protein kinase (CDPK). Pathway analysis showed the carbon assimilation pathway, followed by starch metabolism, to be most perturbed under HS in wheat. A positive correlation was established between the expression of SAAPs at transcript and protein levels in wheat under HS (Kumar et al., 2019).

Adaptive responses to HS also involve various post-translational modifications (PTMs) of proteins. There is a detailed literature on the effects of HS on PTMs such as protein phosphorylation in rice and wheat (Chen et al., 2011; Wu et al., 2014). Currently, >300 PTMs have been detected and partly characterized, and the numbers are increasing (Wu et al., 2016). HS has also been shown to enhance the small ubiquitin-like modification (SUMOylation) of particular proteins (Miller and Vierstra, 2011). SUMOylation of accessible lysines on target proteins is mediated in plants by a sequential three enzyme conjugation pathway: the E1 activating enzyme heterodimer (SAE1a or b combined with SAE2), a single E2 conjugating enzyme (SCE1), and at least two E3 ligases (SIZ1 and MMS21/HPY2). In *Arabidopsis thaliana* L. (Heynh), the activity of the HS TF HSFA2 was shown to be regulated by SUMOylation (Cohen-Peer et al., 2010). Moreover, in potato, rapid changes in protein SUMOylation and serine phosphorylation were observed in response to HS (Colignon et al., 2019). In addition, the overexpression of SlSIZ1 E3 ligase was shown to enhance heat tolerance in tomato (Zhang et al., 2018).

### Metabolomics and lipidomics

Major environmental stresses cause metabolic reorganization towards homeostasis, maintaining essential metabolism and synthesizing metabolites with stress-protective and signaling characteristics. In plants such as tomato (Paupière et al., 2017), maize (Qu et al., 2018), barley (Templer et al., 2017), wheat (Thomason et al., 2018), soybean (Xu et al., 2016), and citrus (Zandalinas et al., 2017), metabolism reprogramming under HS has been studied by untargeted metabolomics. For example, in maize, under CO_2_ stress and a sudden HS, malate, valine, isoleucine, glucose, starch, sucrose, proline, glycine, and serine were successfully found as key players in the combined stress response (Qu et al., 2018). Under both heat and drought stress, amounts of amino acids, and antioxidants such as glutathione and α-tocopherol, were highly affected in two cultivars of barley (Templer et al., 2017); in particular, this combination of stresses led to accumulation of free amino acids in the leaf. In post-anthesis wheat, the amounts of some amino acids increased, while others decreased [l-arginine, l-histidine, l-tryptophan. l-threonine, 4-aminobutanoate (GABA), l-aspartate, and l-phenylalanine]; sugars, sugar alcohols, and other organic compounds were among the major groups of metabolites whose concentration decreased in tissue due to HS.

Thomason et al. (2018) identified the metabolites that exhibited the greatest decreases during HS in wheat: drummondol, anthranilate, dimethylmaleate, galactoglycerol, guanine, and also glycerone. Soybean subjected to both HS and excess CO_2_ had decreased starch, fructose, glucose, sucrose, and maltose concentrations, whereas pinitol increased with temperature (Xu et al., 2016). Zandalinas et al. (2017) found in citrus that water stress and HS, and their combination, altered metabolite levels related to glycolysis and the TCA cycle, the chloroplastic phase of the glyoxylate/dicarboxylate cycle, and the quantity of polar metabolites participating in the phenylpropanoid pathway arising from shikimic acid, depending on the level of stress. In tomato pollen, under HS, most of the putatively identified secondary metabolites belonged to three major groups: flavonoids, polyamines, and alkaloids (Paupière *et al.*, 2017). Other examples come from untargeted metabolomics experiments where it was found that salicylic acid, ascorbic acid, sulfur-containing molecules (glutathione), phenolic secondary metabolites, and in general all antioxidant molecules can reduce HS symptoms in many plant species (Mobin *et al.*, 2017; Feng *et al.*, 2018; Muhlemann *et al.*, 2018; Niu and Xiang, 2018; Salvi et al., 2018; Ihsan *et al.*, 2019). Other major aspects of high temperature stress are cell signaling, molecular transport, and the changes in lipid metabolism as a basic mechanism to regulate membrane fluidity.

Lipids, which are major components of the membranes of cells and organelles, are among the first targets of ROS produced during HS and directly by high temperatures (Narayanan et al., 2016, 2018). Stress-induced lipid peroxidation of unsaturated fatty acids and other changes in membrane lipid profiles can lead to membrane damage, electrolyte leakage, and cell death (Liu and Huang, 2000). Under severe HS and long-term HS conditions, both chloroplastic and extra-plastidial glycerolipids are oxidized, yielding oxylipin-containing glycerolipids and other cytotoxic molecules such as acrolein and methyl vinyl ketone (MVK), derived from peroxidation of trienoic ω-3 fatty acids (Vu et al., 2012).

Accumulation of highly saturated fatty acids helps confer HS tolerance by reducing structural membrane fluidity, which is increased at high temperatures (Escandón et al., 2018). There is an interesting correlation between the type of metabolites involved and the need to protect specific cellular functions or cell compartments from the adverse effects of stress. In addition to acyl oxidation, plants regulate other aspects of membrane lipid composition in response to changes in temperature; in particular, the chloroplast membranes that host the photosynthetic apparatus are thought to be highly vulnerable to damage caused by HS (Welti et al., 2007; Zheng et al., 2011). Severe HS results in increasing frequency of membrane phase separation of non-bilayer-forming glycerolipids. Moderate HS (30–45 °C) results in thylakoid grana membrane destacking (Higashi and Saito, 2019). During HS, the endoplasmic reticulum (ER) is the major site for membrane phosphatidylcholine (PC) synthesis in plants (Jessen et al., 2015). Free fatty acids are exported from chloroplasts and re-esterified to CoA in the cytosol. PC synthesis in the ER utilizes a mixed pool of acyl-CoA substrates via the Kennedy pathway and the PC acyl editing pathway (Lands cycle) (Wang et al., 2012*a*). Fatty acid desaturase 2 (FAD2) converts PC-bound 18:1 to 18:2 in the ER and, during HS, FAD2 mediates polyunsaturation of ER glycerolipids and plays a role in the plant ER stress response to HS (Martinière et al., 2011). Chloroplastic galactolipid biosynthesis through the eukaryotic pathway needs a transfer of glycerolipids from the ER to chloroplasts by phospholipid flippase ALA10 which interacts with FAD2 affecting the degree of PC saturation. LACS4/9 proteins are also involved in this mechanism (Botella et al., 2016).

At the chloroplastic outer and inner membranes, five trigalactosyldiaglycerols (TGDs) and proteins TGD1, 2, 3, 4, and 5 form a transporter complex that mediates ER to plastid lipid trafficking (Xu *et al.*, 2008). However, there is no concerted induction of these membrane proteins at the transcriptional level under HS in Arabidopsis (Higashi et al., 2015). In chloroplasts, long-term HS (>1 d) decreases the levels of glycerolipids containing 16:3 and/or 18:3, and reduces the activity of all FAD enzymes (Higashi and Saito, 2019). In general, during HS and other environmental stresses, conversion of monogalactosyldiacylglycerol (MGDG) into digalactosyldiacylglycerol (DGDG) increases through the action of DGDG synthases, encoded by DGD1 and DGD2. Terrestrial plants increase the ratio of DGDG to MGDG to improve thylakoid membrane stability under various abiotic stresses (Higashi et al., 2018). MGDGs are also decreased through the action of specific lipases (DAD1; PLIP1, 2, and 3; and HIL1). These recent studies suggest that lipases which are localized in chloroplasts have an important role in the lipid remodeling process under HS (Higashi and Saito, 2019).

Glycolipids and phospholipids are converted to triacylglycerol (TAG) in the form of oil bodies, as a transient storage depot for acyl groups prior to membrane lipid recycling/degradation: phospholipid:diacylglycerol acyltransferase 1 (PDAT1) transfers an acyl group from PC to diacylglycerol (DAG), and produces TAG. Mutant *pdat1* seedlings are more sensitive to HS than the wild type; therefore, PDAT1 contributes to HS-induced TAG accumulation, and plays a role in HS response in Arabidopsis seedlings (Mueller et al., 2017). Other chloroplast-localized enzymes that maintain the structure of the thylakoid membranes during HS are FAX 1, 2, and 3, and VIPP1(Zhang et al., 2012; N. Li et al., 2015). HS also changes the lipid composition of the plasma membrane and of the ER; PC and PE (phosphatidylethanolamine) phospholipids are major components of extra-plastidial membranes. A decrease in the PE polyunsaturation level is likely to improve membrane stability under HS (Narayanan et al., 2016). Long-term HS increases phosphatidylserine (PS) content in the leaves of wheat (Narayanan et al., 2016).

Most of the lipidomics investigations of the response to HS have been performed on Arabidopsis, with only a few studies on crops such as wheat and rice. One example of lipidomics applied to HS in wheat found that the decrease in the photosynthetic rate under HS is due to lipid desaturation, oxidation, acylation, and consequent damage to organelles (Djanaguiraman et al., 2018). The membrane protein–lipid associations are of great importance for the maintenance of membrane fluidity; in this context, overexpression of the gene *OsFBN1* (coding for a fibrillin) facilitates the import of lipids to the chloroplast during HS and the consequent grain filling (Djanaguiraman et al., 2018).

It has been suggested that diminishing the ROS pool directly has positive consequences on the state of the lipids. For example, modulating antioxidant defenses and the detoxification of methylglyoxal (MG) through the application of exogenous glutathione to mung bean [*Vigna radiata* (L.) R.Wilczek] increased the tolerance of the plants to HS in the short term (48 h) (Nahar et al., 2015). The increase in certain plant hormones can also be beneficial to the lipid status in rice during HS (Lim et al., 2017). For example, high concentrations of indole-3-acetic acid (IAA) or brassinosteroids can successfully initiate a mechanism for the decrease of primary ROS caused by HS that attack lipid membranes (MGDGs and sulfoquinovosyldiacylglycerols) and thylakoids (Thussagunpanit et al., 2015; Sharma et al., 2018). In a non-targeted lipidomics experiment in rice under both HS and water stress, 298 lipids responded to the changes in conditions; of these, 128 were identified. Interestingly, in the case of HS, a decrease in the unsaturation of lipids was predominant and can be linked to an increase in the cell membranes’ rigidity (Navarro-Reig et al., 2019). In a lipidome-wide study of tomato leaf under HS, changes in 791 molecules were detected between 20 °C and 38 °C. These results indicate that the most important changes at the lipidome-wide level occur in tocopherols, plastoquinone/plastoquinol (and their metabolites), and in the degree of fatty acid saturation of galactolipids (Spicher et al., 2016).

### Epigenetic modifications

The epigenetic changes that occur at the DNA level through methylation of cytosine residues or at the level of chromatin by post-translational modifications of histones can result in altered gene transcription, and are an important mechanism in regulating gene expression during development and in response to environmental stimuli (Zhang and Hsieh, 2013). The persistence of a memory of temperature stress is dependent not only on the persistence of HSPs and their TFs but also on the expression of certain genes relevant for epigenetic signatures (Liu et al., 2015). A primary stress episode can sensitize plants for acclimation to subsequent stresses, resulting in faster or stronger changes in gene expression upon repeated exposure (Vriet et al., 2015), constituting a physiological priming mechanism. This primary event is fundamental in the acquisition of thermotolerance (Sanyal et al., 2018). A comprehensive RNA-Seq (RNA sequencing) analysis of gene expression and splicing events in HS-primed and non-primed plants has been carried out (Ling et al., 2018). This study showed alternative splicing as a novel and significant part of HS priming-induced memory and that this is critical for enhanced stress tolerance. However, most studies have been carried out in Arabidopsis, and in cultivated plants the understanding of the molecular mechanisms involved still represents a challenge (Friedrich et al., 2019).

Further epigenetic control is due to miRNAs, which are emerging as factors involved in transcriptional regulation and HS memory. miRNAs are involved directly in HS adaptation by acting as post-transcriptional regulators. Plant miRNAs bind their target transcripts and cleave and/or degrade the target mRNA molecule (Budak and Akpinar, 2015; Alptekin et al., 2017). The regulation of stress-responsive genes through activation of miRNAs is particularly important under abiotic stress conditions (Liu et al., 2017; Gahlaut et al., 2018).

Experimental validation of miRNA targets is still a challenge in polyploid crops including wheat. Recently, HS-responsive miRNAs have been reported from several susceptible and tolerant cultivars, with responses primarily being invoked within 24 h of treatment (Qin et al., 2008; Kumar et al., 2015). Furthermore, Ravichandran et al. (2019) performed a detailed miRNA and isomiR (miRNA isoforms) annotation and validated the HS-regulated miRNA target genes associated with thermotolerance. They confirmed a high degree of conservation between dicots and monocots for miR156 regulation of squamosa promoter-binding-like protein (Stief et al., 2014), miR159 regulation of MYB transcription factor (Wang et al., 2012*b*), miR166 regulation of homeobox leucine-zipper protein (Arikit et al., 2014), and miR398 regulation of SOD (Jagadeeswaran et al., 2009). New miRNA targets were also identified for organelle-specific transcripts such as pentatricopeptide repeat-containing proteins and mitochondrial transcription termination factor-like proteins.

Interestingly, several miRNAs showed altered expression patterns under heat and cold stresses in wheat, including miR164 (targeting HSP17) and miR319 (targeting a MYB transcription factor). These are up-regulated during a cold stress response but down-regulated in response to HS. On the contrary, miR160 and miR164 were down-regulated in response to heat, resulting in the induction of HSP expression, and up-regulated under cold stress. Regulating mechanisms triggered by high temperature can be reversed by cold stress, although the underlying mechanisms may be similar (Alptekin et al., 2017).

## Breeding for heat tolerance

To ensure food security for future generations, agriculture must double the current crop production rate despite the predicted threats, which include climate change (Sedeek et al., 2019), decrease in arable land and desertification, salinization, and emerging diseases. Plant breeders need affordable and scalable solutions to achieve a more sustainable production. Approaches to reach the essential goal of producing more with less include (i) harnessing natural and artificial mutations; (ii) exploiting the available genetic resources to produce new genetic material more tolerant to HS and related secondary stresses; (iii) improving the ability to screen and identify the available sources of resilience; and (iv) developing new breeding techniques.

### Conventional breeding approach

The effects of HS and prolonged heat waves on food production are heightened by a greater genetic uniformity in crop plants resulting from the narrowing of the varieties used in developed countries (Fu, 2015; Lopes et al., 2015). This has prompted increased efforts to identify new genetic resources and useful traits to mitigate or counteract the effects of climate change on crop productivity (Pignone et al., 2015; Janni et al., 2018) (Table 3).

**Table 3.** Harnessing plant genetic resources for heat stress breeding

Species	Countries of origin	Number of accessions	Source	Type of study	Reference
Barley	Spain and Germany	N/A	Saatzucht Breun GmbH	Gene expression studies	Cantalapiedra et al. (2017)
Chickpea	Several countries	300	N/A	GWAS	Thudi et al. (2014)
Chickpe0a	India	280	ICRISAT	Identification of heat tolerance superior lines	Krishnamurthy et al. (2011)
Maize	N/A	100 inbred lines	N/A	Field trial	Naveed et al. (2016)
Rice	Several countries	455	N/A	QTL identification	Ye et al. (2015)
Rice	Several countries	511	IRRI	Identification of heat tolerance superior lines	Tenorio et al. (2013)
Tomato	Several countries	81	University of Naples (Italy)	GWAS	Ruggieri et al. (2019)
Tomato	N/A	44	TGRC, University of California, Davis	Identification of heat tolerance superior lines	Alsamir et al. (2017)
Wheat	Several countries	1711	CYMMIT	GWAS	Singh et al. (2018)
Wheat	Mexico	2255	CYMMIT	Identification of heat tolerance superior lines	Hede et al. (1999)

CYMMIT, International Maize and Wheat Improvement Center; GWAS, genome-wide association study; ICRISAT, International Crops Research Institute for the Semi-Arid Tropics; IRRI, International Rice Research Institute; N/A not applicable; QTL, quantitative trait locus; TGRC, Tomato Genetics Resource Center

It is well established that responses in crop species to abiotic stress, including HS, form a complex quantitative trait whose inheritance is controlled by a synergy between genes identified as QTLs, distributed throughout the genome. Traditionally QTL mapping has been used to identify new genetic variability and new sources of tolerance for introduction to breeding programs. However, QTL regions can be quite large and may contain many genes to be investigated as potential candidate genes, and many QTL studies had limited value for breeding because of low marker density. More recently, genotyping-by-sequencing (GBS) has allowed an increase in the number of markers, in particular SNPs (single nuclear polymorphisms), evenly distributed throughout the genome (Spindel and Iwata, 2018). In this way, it is now possible to obtain genetic maps with high resolution and precise mapping of QTLs, and in some cases identifying candidate genes controlling associated quantitative traits (Bhat et al., 2016). The application of genome-wide association studies (GWAS) permits narrowing down the candidate regions to explore specific haplotypes in natural populations and even wild species (George and Cavanagh, 2015; Verdeprado et al., 2018).

In wheat, several genomic regions associated with heat tolerance have been mapped using QTL analysis, often combined with GWAS and GBS. Major QTL clusters associated with drought and heat tolerance have been mapped on several chromosomes (Vijayalakshmi et al., 2010; Paliwal et al., 2012; Talukder et al., 2014; Acuña-Galindo et al., 2015; Shirdelmoghanloo et al., 2016; Sharma et al., 2017; Maulana et al., 2018). GWAS of sorghum seedlings under HS has identified associated key genomic regions, and specific alleles from these regions can be used to develop heat-tolerant sorghum cultivars (Chopra et al., 2017). Through inheritance studies, Marfo and Hall (1992) reported two dominant genes controlling most of the heritable tolerance to heat at pod set in cowpea. Four QTLs were identified in cowpea as associated with pod set number per peduncle under HS, and markers were utilized in breeding applications (Lucas et al., 2013; Pottorff et al., 2014). Syntenic analysis of the closely related soybean genome identified HSPs and HSFs in these QTL regions (Liu et al., 2019). In rice, heat tolerance at the flowering stage is controlled by several QTLs that have been used in breeding programs based on gene pyramiding (Ye et al., 2012, 2015; Kilasi et al., 2018).

A major QTL for thermotolerance was successfully identified and cloned in African rice (*Oryza glaberrima* Steud.); *thermo-tolerance 1* (*TT1*) encodes an α2 subunit of the 26S proteasome involved in the degradation of ubiquitinated proteins. The allele *OgTT1* of the thermotolerant accession permitted increased thermotolerance through a more efficient elimination of cytotoxic denatured proteins and effective maintenance of heat response processes than the non-tolerant cultivar carrying the *OsTT1* allele. Overexpression of *OgTT1* was associated with enhanced thermotolerance in rice, Arabidopsis, and *Festuca elata* Keng f. ex E.B.Alexeev (X.M. Li et al., 2015).

In tomato, several QTLs involved in heat tolerance have been identified (Xu et al., 2017), while two QTL hot spots for heat tolerance with respect to grain yield were found in maize (Jha et al., 2014; Frey et al., 2016).

Screening for genotypic differences, and effective selection techniques, are crucial for identifying heat-tolerant parental sources and for inheritance studies, and also for crop improvement by combining other traits which are influenced by heat tolerance (Marfo and Hall, 1992; Hall, 2004). Since photosynthesis and reproductive development processes are directly adversely affected by HS (Prasad et al., 2008), desired characteristics of a heat-tolerant variety will be higher photosynthetic rates (e.g. stay-green leaves), enhanced membrane thermostability, and stable pod set or grain production under high temperature conditions (Bita and Gerats, 2013). Methods for breeding for heat tolerance in cowpea involved selection for abundant flower production and greater pod set under higher night temperatures and long-day growing conditions (Marfo and Hall, 1992). These efforts resulted in release of the HStolerant cowpea variety California Blackeye 27 (CB27) with better yield (Ehlers et al., 2000). This variety was crossed with Pima (a Nigerian heat-tolerant germplasm) giving Apagbaala, a cowpea variety intended for Ghana (Padi et al., 2004). However, this was not heat tolerant in Ghana even though it performed well under Californian growing conditions.

The common bean (*Phaseolus vulgaris* L.) has also been investigated. Initial screening at CIAT (Centro Internacional de Agricultura Tropical) of a germplasm core set for HS tolerance identified 30 resistant lines. Breeding programs utilizing these genetic resources helped to develop heat-tolerant bush (CIAT, 2006) and climbing bean lines (Blair et al., 2006). Moreover, introgression of Tepary bean (*Phaseolus acutifolious* A.Gray) genes into *P. vulgaris* gave an interspecific line that was used as a parent for breeding heat-tolerant lines (Polanía et al., 2017). Recent screening of chickpea genotypes indicated the existence of extensive genotypic variation for reproductive stage heat tolerance; further studies led to the release of heat-tolerant breeding line ICCV 92944 for late-sown conditions (Gaur et al., 2019).

Targeted breeding efforts will help to build heat tolerance in crops (Reynolds et al., 2011). A conceptual model to improve heat tolerance in wheat was proposed involving genetically determined physiological traits such as light interception, radiation use efﬁciency, and partitioning of total assimilates. The physiological breeding approach combines all these traits towards generating a cumulative genetic effect on yield (Cossani and Reynolds, 2012). Data sets from three different spring bread wheat nurseries at CIMMYT (International Maize and Wheat Improvement Center) with different breeding goals were extensively analyzed, and the results showed that spring wheat breeding targeted against abiotic stress delivers better genetic gains in warmer environments (Gourdji et al., 2013). The performance of yield traits under heat and non-stressed environments has been used to identify heat-tolerant genotypes for breeding programs (Ni et al., 2018; Gaur et al., 2019). The identification of critical genes controlling heat tolerance in common wheat led to the release of new cultivars of bread wheat and durum wheat capable of withstanding severe heat (Tadesse et al., 2019), for example the cultivar Faraj, which is able to maintain yield under heat and drought conditions El Hassouni et al. (2019).

It has been shown that compared with direct selection for grain yield, indirect selection through secondary traits with high heritability and significant association with grain yield under stress is a more effective approach in stress tolerance breeding (Bänziger and Lafitte, 1997; Bänziger et al., 2000; Bheemanahalli et al., 2017). In maize, traits associated with reproductive success under heat stress (anthesis–silking interval, pollen viability, stigma receptivity, tassel blast, tassel sterility, and seed set percentage under open pollinated conditions) and other morpho-physiological traits (leaf firing, senescence, and chlorophyll content) were studied along with grain yield for the selection of HS-tolerant germplasms (Alam et al., 2017). As a result, two maize genotypes, VL05728 and VL05799, with better seed setting due to reproductive success under stress were identified as tolerant lines for heat stress (Alam et al., 2017). Several maize breeding programs have successfully increased yields in HS conditions (Cairns and Prasanna, 2018). The identification of suitable donors, highly tolerant to HS and to the combination of HS and drought stress, was successfully achieved by evaluating 300 (Cairns et al., 2013) or 29 (CIMMYT) maize inbred lines (Dinesh et al., 2018). These studies are the best examples to show the significant molecular diversity among maize inbred lines selected for heat tolerance

Reproductive stage HS in rice causes substantial yield loss. Delayed flowering, reduced pollen dispersal, low pollen production, spikelet sterility due to poor anther dehiscence, and impaired starch synthesis during grain development are the major problem areas (Jagadish et al., 2016; Arshad et al., 2017). Natural genetic variation for HS tolerance exists in rice, and significant genetic components controlling these variations have been identified. Several QTLs and genes associated with HS tolerance have been reported (Ye et al., 2015;Shanmugavadivel et al., 2017; Kilasi et al., 2018), and some have been characterized (Krishnan et al., 2011), but more translational research is needed in this area. Guodao 6 and Xieyou 46 are heat-tolerant hybrids developed with stable high rates of grain setting and spikelet fertility under HS (Tao et al., 2008). Other varieties including Fusaotome (tolerant), Hanahikari, Koshijiwase, and Tentakaku (moderately tolerant) were used in breeding for grain quality under heat stress (Ishizaki, 2006). In rice, HS-tolerant traits from line N22 (Jagadish et al., 2008) have been used for introgression into other varieties for developing climate change-ready rice (Ye et al., 2015). QTLs for HS tolerance during the reproductive stage were identified using a recombinant inbred population between N22 and IR64 (Ye et al., 2012).

In tomatoes, reproduction is particularly sensitive to continuous mild heat, which mainly affects pollen viability. The Asian Vegetable Research and Development Center (AVRDC) has identified 39 tolerant lines after several years of natural genetic variation studies. Some of these were used in their tomato breeding programs which produced HS-tolerant lines, such as Equinox (Scott et al., 1995) and Sun Leaper (Gardner, 2000). Another example is the development of ‘Amelia’, a heat-tolerant tomato cultivar for tropical conditions (Gil et al., 2004). Identification of more QTLs associated with heat tolerance in tomatoes (Wen et al., 2019) could enhance breeding efforts for development of stress-tolerant tomato varieties.

The development of molecular tools to accelerate breeding is crucial for the identification of useful traits and their application in breeding programs. In particular, marker-assisted selection (MAS) to improve breeding efficiency has become commonplace. Many MAS strategies have been developed, including marker-assisted backcrossing with foreground and background selection, enrichment of favorable alleles in early generations, and selection for quantitative traits using markers at multiple loci and across multiple cycles of selection (Bassi et al., 2016). MAS requires the use of markers flanking the target locus, and is considered one of the most efficient methods when working on complex traits having a quantitative hereditary characteristic, such as HS tolerance (Tayade et al., 2018). MAS can be used in forward breeding to enrich the allelic frequency for a few desired traits with strong additive QTLs in early selection cycles, to introgress favorable alleles into an elite background, and for integration of (native) traits into a breeding pipeline. This approach has been used in maize to develop improved lines for stress-prone environments (Beyene et al., 2016).

The exploitation of GWAS has led to the approach called genomic selection (GS) a promising tool to design novel breeding programs and to develop new marker-based models for genetic evaluation. The most important factor for its successful and effective implementation in crop species is the availability of genome-wide high-throughput, cost-effective, and flexible molecular markers, having low ascertainment bias, suitable for large population sizes, as well as for both model and non-model crop species with or without the reference genome sequence (Bhat et al., 2016). As a pre-breeding tool, it can serve to identify genetic materials with beneficial variation for complex traits (Wang et al., 2018), predicting the breeding value of an individual within a breeding population. It also provides new opportunities to increase genetic gain of complex traits efficiently. GS has been applied to active breeding programs in several crop species including wheat (Song et al., 2017), rice (Spindel and Iwata, 2018), soybean (Duhnen et al., 2017; Matei et al., 2018), sunflower (Dimitrijevic and Horn, 2017), maize (Cerrudo et al., 2018), chickpea (Roorkiwal et al., 2016), and grapes (Fodor et al., 2014; Viana et al., 2016).

GS and marker-assisted recurrent selection (MARS), widely used in the private sector, are proving efficient for the development of novel cultivars in many crops (Tayade et al., 2018). The major advantage of GS with respect to MAS/MARS is that alleles with minor effects can be captured and used in selection (Cairns and Prasanna, 2018). For both approaches, success depends on excellent phenotypic characterization during the discovery or training phase, respectively.

### New breeding techniques to increase heat tolerance

Genetic modification through biotechnology and other new breeding techniques (NBTs) is a powerful strategy that offers novel opportunities to improve crop adaptability. However, general public concerns and complex legislation are limiting the application of NBTs. Encouraging data collected from genetics can be exploited to significantly increase tolerance to both biotic stresses and abiotic stresses such as salinity, drought, heat, and cold (Zou et al., 2011; Raza et al., 2019). Several studies have used TF genes and other genes associated with abiotic stress tolerance as targets for development of new varieties (Lamaoui et al., 2018). Crops including wheat, maize, tomato, and rice have been genetically modified to enhance thermotolerance, targeting mainly HSPs and HSFs (Fu et al., 2008; Qi et al., 2011; Xue et al., 2015; Casaretto et al., 2016; Wang et al., 2016; Trapero-Mozos et al., 2018). Transgenic cotton plants developed to overexpress an HSP, AtHSP101, in pollen had improved pollen germination and pollen tube growth under high temperature. This significantly enhanced the overall heat tolerance of reproductive tissues and reduced yield losses due to high temperature, supported by both greenhouse and field evaluation of transgenic plants (Burke and Chen, 2015). A survey of the transgenic lines identified to enhance heat tolerance in several species is reported in Table 4.

**Table 4.** List of selected heat stress- (HS) tolerant transgenic plants

Crop	Target gene or protein/sources	Promoter	Stress or trait	Reference
Maize	*OsMYB55*/rice	Maize ubiquitin Ubi1 promoter/overexpression	Increased drought and HS tolerance	Casaretto et al. (2016)
Maize	*ZmNF-YB2*/maize	Rice actin 1 constitutive promoter/overexpression	Enhanced drought tolerance and photosynthetic capacity	Nelson et al. (2007)
Potato	*HSc70* allelic variant/ potato	HSc70 native promoter	Greater tolerance to HS as determined by improved yield	Trapero-Mozos et al. (2018)
Rice	*HSP70*/rice	CaMV 35S	Overexpression manifested enhanced tolerance to HS	Qi et al. (2011)
Rice	*Athsp101* /Arabidopsis	CaMV 35S promoter	Increased tolerance to high temperature	Katiyar-Agarwal et al. (2003)
Rice	*OsRab7*	CaMV 35S	Greater tolerance to HS as determined by improved yield	El-Esawi and Alayafi (2019)
Rice	*TaMBF1c*/wheat	Maize ubiquitin 1	Higher thermotolerance than control plants at both seedling and reproductive stages	Qin *et al.* (2015)
Soybean	*P5CR*/Arabidopsis		Enhanced HS tolerance	De Ronde et al. (2004)
Tobacco *(Nicotiana tabacum* L.)	*HSP70-1*/tobacco	CaMV 35S	Transgenics possessed enhanced tolerance to HT stress	Montero-Barrientos et al. (2008)
Tobacco	*HSP70-1*/brassica		Enhanced tolerance to HT stress	Wang et al. (2016)
Tomato	*HSP21*/tomato	CaMV 35S	Overexpression protected PSII from temperature-dependent oxidative stress; early accumulation of carotenoids noted	Neta-Sharir et al. (2005)
Wheat	*Hsf6A* /wheat	Barley HVA1s promoter/drought inducible, up-regulated	Improved thermotolerance	Xue et al. (2014)
Wheat	*EF-Tu*/maize	Maize ubiquitin 1 promoter/overexpression	Improved thermotolerance	Fu et al. (2008)
Wheat	Sucrose transporter gene *HvSUT1*/barley	Hordein B1 promoter	Increased enhanced in sucrose transport and shows a superior performance for many yield-related traits compared with control	Weichert et al. (2017)
Wheat	*TaHsfA6f*/wheat	HVA1s	Improved thermotolerance	Xue et al. (2015)
Wheat	*TaFER-5B*/wheat	Maize ubiquitin 1	Enhance thermotolerance	Zang et al. (2017)
Wheat	*TaGASR1/*wheat	NA	Improved tolerance to HS and oxidative stress	Zhang et al. (2017)
Wheat	*TaHsfC2a*/wheat	NA	Improved thermotolerance	Hu *et al.* (2018)

The availability of genomic sequences for several crops together with genome editing techniques has opened up new breeding possibilities for almost any given desirable trait (Jaganathan et al., 2018). The most common technique available for genome editing, clustered regularly interspaced palindromic repeats (CRISPR)/CRISPR-associated protein 9 (Cas9), modifies a genome in a targeted manner, and has been used in nearly 20 crop species so far including rice, tomato, potato, cotton, soybean, maize, sorghum, and wheat (Table 5) (Ricroch et al., 2017; Jaganathan et al., 2018; Zaidi et al., 2018). There are at least 13 different patents on new CRISPR/Cas9 approaches, which makes editing even more simple and secure, and new perspectives in the production of novel genetic variability are opened up by its application as an allelic-drive tool, engineering and repairing pathways, and introducing specific point mutations or insertions (Guichard et al., 2019).

**Table 5.** Genome editing approaches used for heat stress breeding

Crop	Target gene	Stress or trait	Reference
Maize	*ARGOS8*	Improved yield under drought stress condition	Shi et al. (2017*a*)
Rice	*OsPDS*, *OsMPK2*, *OsBADH2*	Abiotic stress tolerance	Shan et al. (2013)
Rice	*OsMPK2*, *OsDEP1*	Yield under stress	Shan et al. (2014)
Rice	*GS3*, *Gn1a*	Grain size and number increase	Shen et al. (2017)
Rice	*GW2*, *GW5*, *TGW6*	Grain weight increase	Xu et al. (2016)
Rice	*Gn1a*, *DEP1*, *GS3*	Grain size and number Increase in dense, erect panicles	Li et al. (2016)
Wheat	*TaDREB2*, *TaERF3*	Abiotic stress tolerance	Kim et al. (2018)

Currently, attempts exploiting genome editing to increase heat tolerance target several genes mainly involved in the ethylene response and TFs, with the final aim to increase yield under abiotic stresses including HS (Li et al., 2016; Shi et al., 2017*a*; Kim et al., 2018). However, despite the abundance of novel genetic material generated through CRISPR, few field experiments have been conducted associated with breeding programs.

### Mutational breeding

Mutational breeding strategies have been applied since the late 1990s to generate new variability in plants (Gilliham et al., 2017; Uauy, 2017). An important breakthrough came with the development of the TILLING (targeting induced local lesions in genome) approach, which provides a relatively simple strategy to identify mutations (lesions) in a target sequence independently of their phenotypic effect (Uauy, 2017). TILLING approaches require a population of, typically, ethyl methanesulfonate- (EMS) induced mutants based on the capacity of the chemical agent to generate point mutations distributed randomly in the genome, and a screening method to identify individuals with mutations in the target gene (Wang et al., 2010). TILLING and its updated form, established for polyploidy species through the use of exome capture and the development of the *in silico* TILLING database (Krasileva et al., 2017), have been used extensively to investigate genetic variability in several species mainly targeting quality traits.

Although in TILLING approaches mutagenesis is untargeted and does not provide the versatility of genome editing, crops improved using chemical or radiation mutagenesis via TILLING are not regulated as transgenic organisms in most jurisdictions, increasing their commercial competitiveness with the more precise genome editing approaches (Kumar et al., 2017).

Recently, Comastri et al. (2018) identified a collection of four new small *Hsp26* (*sHsp26*) alleles suitable for enhancing heat tolerance in durum wheat using both *in silico* and *in vivo* TILLING approaches. Following application of TILLING, the ability of a mutated HSP to enhance heat tolerance in tomato has also been demonstrated (Marko et al., 2019). In rice, a TILLING population has been screened for mutations in the HSP genes, and a number of lines showing preliminary enhanced tolerance to HS may be useful for future breeding programs (Yona, 2015; Table 6).

**Table 6.** Mutational breeding in crops

Species	Target trait /genes	Reference
Barley	Starch increases	Sparla et al. (2014)
Bread wheat (*Triticum aestivum* L.)	Starch branching enzyme	Botticella et al. (2011)
Chickpea	Salt tolerance	Kaashyap et al. (2017)
Durum wheat (*Triticum durum* Desf)	Heat stress	Comastri et al., 2018
Durum wheat (*Triticum durum* Desf)	Amylose content	Sestili et al. (2015)
Durum wheat(*Triticum durum* Desf), bread wheat (*Triticum aestivum* L.)	High amylose	Slade et al. (2012)
Oat (*Avena sativa* L.)	Improved β-glucan, antioxidants and omega-3 fatty acid	Chawade et al. (2010)
Peanut	Allergen reduction	Knoll et al. (2011)
Peanut	*LOX* gene	Guo et al. (2015)
Potato	Waxy mutant	Muth et al. (2008)
Rapeseed	Erucic acid synthesis	Wang et al. (2008)
Rapeseed	Sinapine biosynthesis	Harloff et al. (2012)
Sorghum	Reduction of cyanogenic glucosides	Blomstedt et al. (2012)
Sunflower	Accumulation of fatty acids	Kumar et al. (2013)
Tobacco	Leaf yield	Reddy et al. (2012)
Tomato	Fruit biology and ripening	Okabe et al. (2011, 2013)
Tomato	Virus resistance	Piron et al. (2010)
Tomato	Increased pigment and nutrient content	Jones et al. (2012)
Tomato	Early flowering, a solitary flower	MacAlister et al. (2012)
Tomato	Reduced ethylene sensitivity, delayed fruit ripening, prolonged fruit shelf life	Okabe et al. (2011)
Tomato	Decreased ascorbate	Baldet et al. (2013)
Tomato	Decreased carotenoid content	Gady et al. (2012)
Tomato	Increased lycopene content	Silletti et al. (2013)
Tomato	Reduced phenolics content	Di Matteo et al. (2013)

## Conclusions

There are several examples of heat-tolerant varieties in major crops such as wheat, maize, rice, tomato, and legumes successfully developed through conventional breeding. These efforts can be fast-tracked using modern genomic and phenotyping tools. The first HS gene was identified and cloned in tobacco with the pioneering work of Barnett et al. (1979). This gene was shown to encode HSP70, which strongly accumulated in heat-stressed tissue.

Subsequent molecular studies revealed a more complex system with the discovery of HSFs (reviewed in Guo et al., 2016), organelle-specific HSPs (Waters and Vierling, 1999; Waters, 2013), and linkage between HS and the induction of an alternative splicing system (Ling et al., 2018) capable of directing *de novo* HSP synthesis in conditions where most proteins were either produced abnormally or were degraded (Vierling, 1991), remodeling the global protein machinery in cells. However, neither these discoveries nor the networking of all the known elements have produced a satisfying picture of the plant heat stress response. QTLs for heat tolerance offer a different, potentially complementary, interpretation with a stronger emphasis on physiology of reproduction in heat stress conditions.

Current data indicate that at least two different genetic systems can protect plants from the otherwise lethal effects of heat stress: (i) a series of Mendelian HS genes acting possibly in a dominant or semi-dominant way; and (ii) a small number of QTLs which allow plant growth and reproduction under different stress conditions. There has been little progress using transgenic approaches in studies of model plant to crop plant translational genetics. Extensive knowledge has become available on physiological, biochemical, and molecular regulation through advanced phenotyping and multi-omics tools developed over the last decade. All of this suggests that significant advances in crop improvement will be achieved, resulting in the development of new high-yielding varieties with greater heat tolerance and adaptation to climate change.

## Author contributions

The authors contributed equally to the preparation of the review. MJ took on the responsibility of connecting all the different tasks distributed as follows: EM, gene ontology and system biology; MM, protein and metabolites; MG, gene expression and epigenetic modification; BV, gene expression and breeding; and MJ, induction of HS response; NM and HTN coordinated the planning and the writing of the review.

## References

[CIT0001] Acuña-Galindo MA, Mason RE, Subramanian NK, Hays DB 2015 Meta-analysis of wheat QTL regions associated with adaptation to drought and heat stress. Crop Science 55, 477–492.

[CIT0002] Alam MA, Seetharam K, Zaidi PH, Dinesh A, Vinayan MT, Nath UK 2017 Dissecting heat stress tolerance in tropical maize (*Zea mays* L.). Field Crops Research 204, 110–119.

[CIT0003] Almeselmani M, Deshmukh P, Sairam R 2009 High temperature stress tolerance in wheat genotypes: role of antioxidant defence enzymes. Acta Agronomica Hungarica 57, 1–14.

[CIT0004] Almeselmani M, Deshmukh PS, Sairam RK, Kushwaha SR, Singh TP 2006 Protective role of antioxidant enzymes under high temperature stress. Plant Science 171, 382–388. 22980208 10.1016/j.plantsci.2006.04.009

[CIT0005] Alptekin B, Langridge P, Budak H 2017 Abiotic stress miRNomes in the Triticeae. Functional & Integrative Genomics 17, 145–170. 27665284 10.1007/s10142-016-0525-9 PMC5383695

[CIT0006] Alsamir M, Ahmad NM, Keitel C, Mahmood T, Trethowan R 2017 Identification of high-temperature tolerant and agronomically viable tomato (*S. lycopersicum*) genotypes from a diverse germplasm collection. Advances in Crop Science and Technology 5, 1000299.

[CIT0007] Altschuler M, Mascarenhas JP 1982 Heat shock proteins and effects of heat shock in plants. Plant Molecular Biology 1, 103–115. 24317892 10.1007/BF00024974

[CIT0008] Arce D, Spetale F, Krsticevic F, Cacchiarelli P, Las Rivas J, Ponce S, Pratta G, Tapia E 2018 Regulatory motifs found in the small heat shock protein (sHSP) gene family in tomato. BMC Genomics 19, 860. 30537925 10.1186/s12864-018-5190-z PMC6288846

[CIT0009] Arikit S, Xia R, Kakrana A, et al. 2014 An atlas of soybean small RNAs identifies phased siRNAs from hundreds of coding genes. The Plant Cell 26, 4584–4601. 25465409 10.1105/tpc.114.131847 PMC4311202

[CIT0010] Arshad MS, Farooq M, Asch F, Krishna JSV, Prasad PVV, Siddique KHM 2017 Thermal stress impacts reproductive development and grain yield in rice. Plant Physiology and Biochemistry 115, 57–72. 28324683 10.1016/j.plaphy.2017.03.011

[CIT0011] Asthir B, Koundal A, Bains NS 2012 Putrescine modulates antioxidant defense response in wheat under high temperature stress. Biologia Plantarum 56, 757–761.

[CIT0012] Baldet P, Bres C, Okabe Y, Mauxion J-P, Just D, Bournonville C, Ferrand C, Mori K, Ezura H, Rothan C 2013 Investigating the role of vitamin C in tomato through TILLING identification of ascorbate-deficient tomato mutants. Plant Biotechnology 30, 309–314.

[CIT0013] Bänziger M, Edmeades GO, Beck DL, Bellon MR 2000 Breeding for drought and nitrogen stress tolerance in maize: from theory to practice. Mexico: CIMMYT.

[CIT0014] Bänziger M, Lafitte HR 1997 Efficiency of secondary traits for improving maize for low-nitrogen target environments. Crop Science 37, 1110–1117.

[CIT0015] Barghi SS, Mostafaii H, Peighami F, Zakaria RA 2012 Path analysis of yield and its components in lentil under end season heat condition. International Journal of Agriculture: Research and Review 2, 969–974.

[CIT0016] Barnabás B, Jäger K, Fehér A 2008 The effect of drought and heat stress on reproductive processes in cereals. Plant, Cell & Environment 31, 11–38. 10.1111/j.1365-3040.2007.01727.x 17971069

[CIT0017] Barnett T, Altschuler M, McDaniel CN, Mascarenhas JP 1979 Heat shock induced proteins in plant cells. Genesis 1, 331–340.

[CIT0018] Bassi FM, Bentley AR, Charmet G, Ortiz R, Crossa J 2016 Breeding schemes for the implementation of genomic selection in wheat (*Triticum* spp.). Plant Science 242, 23–36. 26566822 10.1016/j.plantsci.2015.08.021

[CIT0019] Berz J, Simm S, Schuster S, Scharf KD, Schleiff E, Ebersberger I 2019 HEATSTER: a database and web server for identification and classification of heat stress transcription factors in plants. Bioinformatics and Biology Insights 13, 1177932218821365. 30670918 10.1177/1177932218821365 PMC6327235

[CIT0020] Beyene Y, Semagn K, Mugo S, et al. 2016 Performance and grain yield stability of maize populations developed using marker-assisted recurrent selection and pedigree selection procedures. Euphytica 208, 285–297. 27397932 10.1007/s10681-015-1590-1 PMC4913958

[CIT0021] Bhat JA, Ali S, Salgotra RK, et al. 2016 Genomic selection in the era of next generation sequencing for complex traits in plant breeding. Frontiers in Genetics 7, 221. 28083016 10.3389/fgene.2016.00221 PMC5186759

[CIT0022] Bhattacharjee S 2012 An inductive pulse of hydrogen peroxide pretreatment restores redox-homeostasis and oxidative membrane damage under extremes of temperature in two rice cultivars. Plant Growth Regulation 68, 395–410.

[CIT0023] Bhattacharya A 2019 Effect of high temperature on crop productivity and metabolism of macro molecules. London: Academic Press.

[CIT0024] Bheemanahalli R, Sathishraj R, Manoharan M, Sumanth HN, Muthurajan R, Ishimaru T, Krishna JS 2017 Is early morning flowering an effective trait to minimize heat stress damage during flowering in rice? Field Crops Research 203, 238–242. 28260830 10.1016/j.fcr.2016.11.011 PMC5310116

[CIT0025] Bita CE, Gerats T 2013 Plant tolerance to high temperature in a changing environment: scientific fundamentals and production of heat stress-tolerant crops. Frontiers in Plant Science 4, 273. 23914193 10.3389/fpls.2013.00273 PMC3728475

[CIT0026] Bita CE, Zenoni S, Vriezen WH, Mariani C, Pezzotti M, Gerats T 2011 Temperature stress differentially modulates transcription in meiotic anthers of heat-tolerant and heat-sensitive tomato plants. BMC Genomics 12, 384. 21801454 10.1186/1471-2164-12-384 PMC3162933

[CIT0027] Blair MW, Iriarte G, Beebe S 2006 QTL analysis of yield traits in an advanced backcross population derived from a cultivated Andean × wild common bean (*Phaseolus vulgaris* L.) cross. Theoretical and Applied Genetics 112, 1149–1163. 16432734 10.1007/s00122-006-0217-2

[CIT0028] Blomstedt CK, Gleadow RM, O’Donnell N, et al. 2012 A combined biochemical screen and TILLING approach identifies mutations in *Sorghum bicolor* L. Moench resulting in acyanogenic forage production. Plant Biotechnology Journal 10, 54–66. 21880107 10.1111/j.1467-7652.2011.00646.x

[CIT0029] Botella C, Sautron E, Boudiere L, Michaud M, Dubots E, Yamaryo-Botté Y, Albrieux C, Marechal E, Block MA, Jouhet J 2016 ALA10, a phospholipid flippase, controls FAD2/FAD3 desaturation of phosphatidylcholine in the ER and affects chloroplast lipid composition in *Arabidopsis thaliana*. Plant Physiology 170, 1300–1314. 26620528 10.1104/pp.15.01557 PMC4775126

[CIT0030] Botticella E, Sestili F, Hernandez-Lopez A, Phillips A, Lafiandra D 2011 High resolution melting analysis for the detection of EMS induced mutations in wheat Sbella genes. BMC Plant Biology 11, 156. 22074448 10.1186/1471-2229-11-156 PMC3228712

[CIT0031] Budak H, Akpinar BA 2015 Plant miRNAs: biogenesis, organization and origins. Functional & Integrative Genomics 15, 523–531. 26113396 10.1007/s10142-015-0451-2

[CIT0032] Burke JJ, Chen J 2015 Enhancement of reproductive heat tolerance in plants. PLoS One 10, e0122933. 25849955 10.1371/journal.pone.0122933 PMC4388472

[CIT0033] Cairns JE, Crossa J, Zaidi PH, et al. 2013 Identification of drought, heat, and combined drought and heat tolerant donors in maize. Crop Science 53, 1335.

[CIT0034] Cairns JE, Prasanna BM 2018 Developing and deploying climate-resilient maize varieties in the developing world. Current Opinion in Plant Biology 45, 226–230. 29779966 10.1016/j.pbi.2018.05.004 PMC6250980

[CIT0035] Cantalapiedra CP, García-Pereira MJ, Gracia MP, Igartua E, Casas AM, Contreras-Moreira B 2017 Large differences in gene expression responses to drought and heat stress between elite barley cultivar Scarlett and a Spanish landrace. Frontiers in Plant Science 8, 647. 28507554 10.3389/fpls.2017.00647 PMC5410667

[CIT0036] Casaretto JA, El-Kereamy A, Zeng B, Stiegelmeyer SM, Chen X, Bi YM, Rothstein SJ 2016 Expression of OsMYB55 in maize activates stress-responsive genes and enhances heat and drought tolerance. BMC Genomics 17, 312. 27129581 10.1186/s12864-016-2659-5 PMC4850646

[CIT0037] Cerrudo D, Cao S, Yuan Y, Martinez C, Suarez EA, Babu R, Zhang X, Trachsel S 2018 Genomic selection outperforms marker assisted selection for grain yield and physiological traits in a maize doubled haploid population across water treatments. Frontiers in Plant Science 9, 366. 29616072 10.3389/fpls.2018.00366 PMC5869257

[CIT0038] Chakraborty K, Bishi SK, Singh AL, Zala PV, Mahatma MK, Kalariya KA, Jat RA 2018 Rapid induction of small heat shock proteins improves physiological adaptation to high temperature stress in peanut. Journal of Agronomy and Crop Science 204, 285–297.

[CIT0039] Chakraborty S, Newton AC 2011 Climate change, plant diseases and food security: an overview. Plant Pathology 60, 2–14.

[CIT0040] Chakraborty U, Pradhan D 2011 High temperature-induced oxidative stress in *Lens culinaris*, role of antioxidants and amelioration of stress by chemical pre-treatments. Journal of Plant Interactions 6, 43–52.

[CIT0041] Chao Y-Y, Hsu YT, Kao CH 2008 Involvement of glutathione in heat shock- and hydrogen peroxide-induced cadmium tolerance of rice (*Oryza sativa* L.) seedlings. Plant and Soil 318, 37.

[CIT0042] Chaturvedi P, Doerfler H, Jegadeesan S, Ghatak A, Pressman E, Castillejo MA, Wienkoop S, Egelhofer V, Firon N, Weckwerth W 2015 Heat-treatment-responsive proteins in different developmental stages of tomato pollen detected by targeted mass accuracy precursor alignment (tMAPA). Journal of Proteome Research 14, 4463–4471. 26419256 10.1021/pr501240n

[CIT0043] Chawade A, Sikora P, Bräutigam M, Larsson M, Vivekanand V, Nakash MA, Chen T, Olsson O 2010 Development and characterization of an oat TILLING-population and identification of mutations in lignin and β-glucan biosynthesis genes. BMC Plant Biology 10, 86. 20459868 10.1186/1471-2229-10-86 PMC3017761

[CIT0044] Chen B, Feder ME, Kang L 2018 Evolution of heat-shock protein expression underlying adaptive responses to environmental stress. Molecular Ecology 27, 3040–3054. 29920826 10.1111/mec.14769

[CIT0045] Chen X, Zhang W, Zhang B, Zhou J, Wang Y, Yang Q, Ke Y, He H 2011 Phosphoproteins regulated by heat stress in rice leaves. Proteome Science 9, 37. 21718517 10.1186/1477-5956-9-37 PMC3150237

[CIT0046] Cho R 2018 How climate change will alter our food. State of the Planet. New York: Columbia University Press.

[CIT0047] Chopra R, Burow G, Burke JJ, Gladman N, Xin Z 2017 Genome-wide association analysis of seedling traits in diverse *Sorghum germplasm* under thermal stress. BMC Plant Biology 17, 12. 28086798 10.1186/s12870-016-0966-2 PMC5237230

[CIT0048] Chou TS, Chao YY, Kao CH 2012 Involvement of hydrogen peroxide in heat shock- and cadmium-induced expression of ascorbate peroxidase and glutathione reductase in leaves of rice seedlings. Journal of Plant Physiology 169, 478–486. 22196946 10.1016/j.jplph.2011.11.012

[CIT0049] Chung E, Kim KM, Lee JH 2013 Genome-wide analysis and molecular characterization of heat shock transcription factor family in *Glycine max*. Journal of Genetics and Genomics 40, 127–135. 23522385 10.1016/j.jgg.2012.12.002

[CIT0050] CIAT 2006 Annual Report of the International Center for Tropical Agriculture (CIAT) https://cgspace.cgiar.org/bitstream/handle/10568/73453/CIAT_Annual_Report_2015-2016_Synthesis.pdf?sequence=6

[CIT0051] Cohen-Peer R, Schuster S, Meiri D, Breiman A, Avni A 2010 Sumoylation of Arabidopsis heat shock factor A2 (HsfA2) modifies its activity during acquired thermotholerance. Plant Molecular Biology 74, 33–45. 20521085 10.1007/s11103-010-9652-1

[CIT0052] Colignon B, Delaive E, Dieu M, Demazy C, Muhovski Y, Antoine A, Raes M, Mauro S 2019 Dual coordination of the SUMOylation and phosphorylation pathways during the response to heat stress in *Solanum tuberosum*. Environmental and Experimental Botany 162, 192–200.

[CIT0053] Comastri A, Janni M, Simmonds J, Uauy C, Pignone D, Nguyen HT, Marmiroli N 2018 Heat in wheat: exploit reverse genetic techniques to discover new alleles within the *Triticum durum* sHsp26 family. Frontiers in Plant Science 9, 1337. 30283469 10.3389/fpls.2018.01337 PMC6156267

[CIT0054] Cossani CM, Reynolds MP 2012 Physiological traits for improving heat tolerance in wheat. Plant Physiology 160, 1710–1718. 23054564 10.1104/pp.112.207753 PMC3510104

[CIT0055] Das S, Krishnan P, Mishra V, Kumar R, Ramakrishnan B, Singh NK 2015 Proteomic changes in rice leaves grown under open field high temperature stress conditions. Molecular Biology Reports 42, 1545–1558. 26323334 10.1007/s11033-015-3923-5

[CIT0056] De Ronde JA, Cress WA, Krüger GHJ, Strasser RJ, Van Staden J 2004 Photosynthetic response of transgenic soybean plants, containing an Arabidopsis P5CR gene, during heat and drought stress. Journal of Plant Physiology 161, 1211–1224. 15602813 10.1016/j.jplph.2004.01.014

[CIT0056a] Debaeke P, Casadebaig P, Flenet F, Langlade N 2017 Sunflower crop and climate change: vulnerability, adaptation, and mitigation potential from case-studies in Europe. OCL 24, D102.

[CIT0057] Devasirvatham V, Tan D 2018 Impact of high temperature and drought stresses on chickpea production. Agronomy 8, 145.

[CIT0058] Di Matteo A, Ruggieri V, Sacco A, Rigano MM, Carriero F, Bolger A, Fernie AR, Frusciante L, Barone A 2013 Identification of candidate genes for phenolics accumulation in tomato fruit. Plant Science 205–206, 87–96. 10.1016/j.plantsci.2013.02.001 23498866

[CIT0059] Dimitrijevic A, Horn R 2017 Sunflower hybrid breeding: from markers to genomic selection. Frontiers in Plant Science 8, 2238. 29387071 10.3389/fpls.2017.02238 PMC5776114

[CIT0060] Dinesh A, Patil A, Zaidi PH, Kuchanur PH, Vinayan MT, Seetharam K 2018 Genetic diversity, linkage disequilibrium and population structure among CIMMYT maize inbred lines, selected for heat tolerance study. Maydica 61, 7.

[CIT0061] Djanaguiraman M, Boyle DL, Welti R, Jagadish SVK, Prasad PVV 2018 Decreased photosynthetic rate under high temperature in wheat is due to lipid desaturation, oxidation, acylation, and damage of organelles. BMC Plant Biology 18, 55. 29621997 10.1186/s12870-018-1263-z PMC5887265

[CIT0062] Dong X, Yi H, Lee J, Nou IS, Han CT, Hur Y 2015 Global gene-expression analysis to identify differentially expressed genes critical for the heat stress response in *Brassica rapa*. PLoS One 10, e0130451. 26102990 10.1371/journal.pone.0130451 PMC4477974

[CIT0063] Driedonks N, Rieu I, Vriezen WH 2016 Breeding for plant heat tolerance at vegetative and reproductive stages. Plant Reproduction 29, 67–79. 26874710 10.1007/s00497-016-0275-9 PMC4909801

[CIT0064] Du Z, Zhou X, Ling Y, Zhang Z, Su Z 2010 agriGO: a GO analysis toolkit for the agricultural community. Nucleic Acids Research 38, W64–W70. 20435677 10.1093/nar/gkq310 PMC2896167

[CIT0065] Duhnen A, Gras A, Teyssèdre S, Romestant M, Claustres B, Daydé J, Mangin B 2017 Genomic selection for yield and seed protein content in soybean: a study of breeding program data and assessment of prediction accuracy. Crop Science 57, 1325.

[CIT0066] El-Esawi MA, Alayafi AA 2019 Overexpression of rice Rab7 gene improves drought and heat tolerance and increases grain yield in rice (*Oryza sativa* L.). Genes 10, E56. 30658457 10.3390/genes10010056 PMC6357162

[CIT0067] Elferjani R, Soolanayakanahally R 2018 Canola responses to drought, heat, and combined stress: shared and specific effects on carbon assimilation, seed yield, and oil composition. Frontiers in Plant Science 9, 1224. 30214451 10.3389/fpls.2018.01224 PMC6125602

[CIT0068] Ehlers JD, Hall AE, Patel PN, Roberts PA, Matthews WC 2000 Registration of ‘California Blackeye 27’ cowpea. Crop Science 40, 854–855.

[CIT0069] El Hassouni K, Belkadi B, Filali-Maltouf A, Tidiane-Sall A, Al-Abdallat A, Nachit M, Bassi FM 2019 Loci controlling adaptation to heat stress occurring at the reproductive stage in durum wheat. Agromomy 9, 414.

[CIT0070] Escandón M, Meijón M, Valledor L, Pascual J, Pinto G, Cañal MJ 2018 Metabolome integrated analysis of high-temperature response in *Pinus radiata*. Frontiers in Plant Science 9, 485. 29719546 10.3389/fpls.2018.00485 PMC5914196

[CIT0071] Fahad S, Bajwa AA, Nazir U, et al. 2017 Crop production under drought and heat stress: plant responses and management options. Frontiers in Plant Science 8, 1147. 28706531 10.3389/fpls.2017.01147 PMC5489704

[CIT0072] Fang C, Dou L, Liu Y, Yu J, Tu J 2018 Heat stress-responsive transcriptome analysis in heat susceptible and tolerant rice by high-throughput sequencing. Ecological Genetics and Genomics 6, 33–40.

[CIT0073] Feng B, Zhang C, Chen T, Zhang X, Tao L, Fu G 2018 Salicylic acid reverses pollen abortion of rice caused by heat stress. BMC Plant Biology 18, 245. 30340520 10.1186/s12870-018-1472-5 PMC6194599

[CIT0074] Fodor A, Segura V, Denis M, Neuenschwander S, Fournier-Level A, Chatelet P, Homa FA, Lacombe T, This P, Le Cunff L 2014 Genome-wide prediction methods in highly diverse and heterozygous species: proof-of-concept through simulation in grapevine. PLoS One 9, e110436. 25365338 10.1371/journal.pone.0110436 PMC4217727

[CIT0075] Fragkostefanakis S, Simm S, Paul P, Bublak D, Scharf KD, Schleiff E 2015 Chaperone network composition in *Solanum lycopersicum* explored by transcriptome profiling and microarray meta-analysis. Plant, Cell & Environment 38, 693–709. 10.1111/pce.12426 25124075

[CIT0076] Frank G, Pressman E, Ophir R, Althan L, Shaked R, Freedman M, Shen S, Firon N 2009 Transcriptional profiling of maturing tomato (*Solanum lycopersicum* L.) microspores reveals the involvement of heat shock proteins, ROS scavengers, hormones, and sugars in the heat stress response. Journal of Experimental Botany 60, 3891–3908. 19628571 10.1093/jxb/erp234 PMC2736902

[CIT0077] Frey FP, Presterl T, Lecoq P, Orlik A, Stich B 2016 First steps to understand heat tolerance of temperate maize at adult stage: identification of QTL across multiple environments with connected segregating populations. Theoretical and Applied Genetics 129, 945–961. 26886101 10.1007/s00122-016-2674-6 PMC4835532

[CIT0078] Frey FP, Urbany C, Hüttel B, Reinhardt R, Stich B 2015 Genome-wide expression profiling and phenotypic evaluation of European maize inbreds at seedling stage in response to heat stress. BMC Genomics 16, 123. 25766122 10.1186/s12864-015-1282-1 PMC4347969

[CIT0079] Friedrich T, Faivre L, Bäurle I, Schubert D 2019 Chromatin-based mechanisms of temperature memory in plants. Plant, Cell & Environment 42, 762–770. 10.1111/pce.13373 29920687

[CIT0080] Fu J, Momcilović I, Clemente TE, Nersesian N, Trick HN, Ristic Z 2008 Heterologous expression of a plastid EF-Tu reduces protein thermal aggregation and enhances CO_2_ fixation in wheat (*Triticum aestivum*) following heat stress. Plant Molecular Biology 68, 277–288. 18622733 10.1007/s11103-008-9369-6

[CIT0081] Fu YB 2015 Understanding crop genetic diversity under modern plant breeding. Theoretical and Applied Genetics 128, 2131–2142. 26246331 10.1007/s00122-015-2585-y PMC4624815

[CIT0082] Gady ALF, Vriezen WH, Van de Wal MHBJ, Huang P, Bovy AG, Visser RGF, Bachem CWB 2012 Induced point mutations in the phytoene synthase 1 gene cause differences in carotenoid content during tomato fruit ripening. Molecular Breeding 29, 801–812. 22408384 10.1007/s11032-011-9591-9 PMC3285762

[CIT0083] Gahlaut V, Baranwal VK, Khurana P 2018 miRNomes involved in imparting thermotolerance to crop plants. 3 Biotech 8, 497. 10.1007/s13205-018-1521-7 PMC6261126 30498670

[CIT0084] Gammulla CG, Pascovici D, Atwell BJ, Haynes PA 2010 Differential metabolic response of cultured rice (*Oryza sativa*) cells exposed to high- and low-temperature stress. Proteomics 10, 3001–3019. 20645384 10.1002/pmic.201000054

[CIT0085] Gardner RG 2000 ‘Sun Leaper’, a hybrid tomato, and its parent, NC HS-1. HortScience 35, 960–961.

[CIT0086] Gaur PM, Samineni S, Thudi M, et al. 2019 Integrated breeding approaches for improving drought and heat adaptation in chickpea (*Cicer arietinum* L.). Plant Breeding 138, 389–400.

[CIT0087] George AW, Cavanagh C 2015 Genome-wide association mapping in plants. Theoretical and Applied Genetics 128, 1163–1174. 25800009 10.1007/s00122-015-2497-x

[CIT0088] Gil MA, López CM, Cuadra MED, Sánchez JA, Coca BM, Martínez SP, Zueco JC 2004 ‘Amalia’: a medium-fruit-size, heat-tolerant tomato cultivar for tropical conditions. HortScience 39, 1503–1504.

[CIT0089] Gilliham M, Able JA, Roy SJ 2017 Translating knowledge about abiotic stress tolerance to breeding programmes. The Plant Journal 90, 898–917. 27987327 10.1111/tpj.13456

[CIT0090] Gillman JD, Biever JJ, Ye S, Spollen WG, Givan SA, Lyu Z, Joshi T, Smith JR, Fritschi FB 2019 A seed germination transcriptomic study contrasting two soybean genotypes that differ in terms of their tolerance to the deleterious impacts of elevated temperatures during seed fill. BMC Research Notes 12, 522. 31426836 10.1186/s13104-019-4559-7 PMC6700996

[CIT0091] Ginzberg I, Barel G, Ophir R, Tzin E, Tanami Z, Muddarangappa T, de Jong W, Fogelman E 2009 Transcriptomic profiling of heat-stress response in potato periderm. Journal of Experimental Botany 60, 4411–4421. 19752048 10.1093/jxb/erp281

[CIT0092] González-Schain N, Dreni L, Lawas LM, Galbiati M, Colombo L, Heuer S, Jagadish KS, Kater MM 2016 Genome-wide transcriptome analysis during anthesis reveals new insights into the molecular basis of heat stress responses in tolerant and sensitive rice varieties. Plant & Cell Physiology 57, 57–68. 26561535 10.1093/pcp/pcv174

[CIT0093] Gourdji SM, Mathews KL, Reynolds M, Crossa J, Lobell DB 2013 An assessment of wheat yield sensitivity and breeding gains in hot environments. Proceedings of the Royal Society B: Biological Sciences 280, 20122190. 10.1098/rspb.2012.2190 PMC3574297 23222442

[CIT0094] Govindaraj M, Pattanashetti SK, Patne N, Kanatti AA 2018 Breeding cultivars for heat stress tolerance in staple food crops. In: Çiftçi YÖ, ed. Next generation plant breeding. InTechOpen.

[CIT0095] Greer DH, Weedon MM 2013 The impact of high temperatures on *Vitis vinifera* cv. Semillon grapevine performance and berry ripening. Frontiers in Plant Science 4, 491. 24348494 10.3389/fpls.2013.00491 PMC3848316

[CIT0096] Guichard A, Haque T, Bobik M, Xu XS, Klanseck C, Kushwah RBS, Berni M, Kaduskar B, Gantz VM, Bier E 2019 Efficient allelic-drive in Drosophila. Nature Communications 10, 1640. 10.1038/s41467-019-09694-w PMC6456580 30967548

[CIT0097] Guo Y, Abernathy B, Zeng Y, Ozias-Akins P 2015 TILLING by sequencing to identify induced mutations in stress resistance genes of peanut (*Arachis hypogaea*). BMC Genomics 16, 157. 25881128 10.1186/s12864-015-1348-0 PMC4369367

[CIT0098] Guo M, Liu JH, Ma X, Luo DX, Gong ZH, Lu MH 2016 The plant heat stress transcription factors (HSFs): structure, regulation, and function in response to abiotic stresses. Frontiers in Plant Science 7, 114. 26904076 10.3389/fpls.2016.00114 PMC4746267

[CIT0099] Gupta OP, Mishra V, Singh NK, Tiwari R, Sharma P, Gupta RK, Sharma I 2015 Deciphering the dynamics of changing proteins of tolerant and intolerant wheat seedlings subjected to heat stress. Molecular Biology Reports 42, 43–51. 25218843 10.1007/s11033-014-3738-9

[CIT0100] Hall AE 2004 Breeding for adaptation to drought and heat in cowpea. European Journal of Agronomy 21, 447–454.

[CIT0101] Han F, Chen H, Li X-J, Yang M-F, Liu G-S, Shen S-H 2009 A comparative proteomic analysis of rice seedlings under various high-temperature stresses. Biochimica et Biophysica Acta 1794, 1625–1634. 19635594 10.1016/j.bbapap.2009.07.013

[CIT0102] Hancock RD, Morris WL, Ducreux LJM, et al. 2014 Physiological, biochemical and molecular responses of the potato (*Solanum tuberosum* L.) plant to moderately elevated temperature: systems biology of heat stress in potato. Plant, Cell & Environment 37, 439–450. 10.1111/pce.12168 23889235

[CIT0103] Harloff H-J, Lemcke S, Mittasch J, Frolov A, Wu JG, Dreyer F, Leckband G, Jung C 2012 A mutation screening platform for rapeseed (*Brassica napus* L.) and the detection of sinapine biosynthesis mutants. Theoretical and Applied Genetics 124, 957–969. 22198204 10.1007/s00122-011-1760-z

[CIT0104] Hasanuzzaman M, Nahar K, Alam MM, Roychowdhury R, Fujita M 2013 Physiological, biochemical, and molecular mechanisms of heat stress tolerance in plants. International Journal of Molecular Sciences 14, 9643–9684. 23644891 10.3390/ijms14059643 PMC3676804

[CIT0105] Hede AR, Skovmand B, Reynolds MP, Crossa J, Vilhelmsen AL, Stølen O 1999 Evaluating genetic diversity for heat tolerance traits in Mexican wheat landraces. Genetic Resources and Crop Evolution 46, 37–45.

[CIT0106] Hewezi T, Léger M, Gentzbittel L 2008 A comprehensive analysis of the combined effects of high light and high temperature stresses on gene expression in sunflower. Annals of Botany 102, 127–140. 18477560 10.1093/aob/mcn071 PMC2712429

[CIT0107] Higashi Y, Okazaki Y, Myouga F, Shinozaki K, Saito K 2015 Landscape of the lipidome and transcriptome under heat stress in *Arabidopsis thaliana*. Scientific Reports 5, 10533. 26013835 10.1038/srep10533 PMC4444972

[CIT0108] Higashi Y, Okazaki Y, Takano K, Myouga F, Shinozaki K, Knoch E, Fukushima A, Saito K 2018 *HEAT INDUCIBLE LIPASE1* remodels chloroplastic monogalactosyldiacylglycerol by liberating α-linolenic acid in Arabidopsis leaves under heat stress. The Plant Cell 30, 1887–1905. 29967047 10.1105/tpc.18.00347 PMC6139690

[CIT0109] Higashi Y, Saito K 2019 Lipidomic studies of membrane glycerolipids in plant leaves under heat stress. Progress in Lipid Research 75, 100990. 31442527 10.1016/j.plipres.2019.100990

[CIT0109a] Hu X-J, Chen D, Lynne Mclntyre C, Fernanda Dreccer M, Zhang Z-B, Drenth J, Kalaipandian S, Chang H, Xue G-P 2018 Heat shock factor C2a serves as a proactive mechanism for heat protection in developing grains in wheat via an ABA-mediated regulatory pathway. Plant, Cell & Environment 41, 79–98. 10.1111/pce.12957 28370204

[CIT0110] Hu X, Wu L, Zhao F, Zhang D, Li N, Zhu G, Li C, Wang W 2015 Phosphoproteomic analysis of the response of maize leaves to drought, heat and their combination stress. Frontiers in Plant Science 6, 298. 25999967 10.3389/fpls.2015.00298 PMC4419667

[CIT0111] Hurkman WJ, McCue KF, Altenbach SB, et al. 2003 Effect of temperature on expression of genes encoding enzymes for starch biosynthesis in developing wheat endosperm. Plant Science 164, 873–881.

[CIT0112] Hurkman WJ, Tanaka CK, Vensel WH, Thilmony R, Altenbach SB 2013 Comparative proteomic analysis of the effect of temperature and fertilizer on gliadin and glutenin accumulation in the developing endosperm and flour from *Triticum aestivum* L. cv. Butte 86. Proteome Science 11, 8. 23432757 10.1186/1477-5956-11-8 PMC3599944

[CIT0113] Ihsan MZ, Daur I, Alghabari F, Alzamanan S, Rizwan S, Ahmad M, Waqas M, Shafqat W 2019 Heat stress and plant development: role of sulphur metabolites and management strategies. Acta Agriculturae Scandinavica, Section B—Soil & Plant Science 69, 332–342.

[CIT0114] Ishizaki K 2006 Evaluation of various screening systems for high grain quality in rice cultivars under high-temperature grain-filling conditions, and the selection of their standard cultivars. Japanese Journal of Crop Science 75, 502–506.

[CIT0115] Jagadeeswaran G, Saini A, Sunkar R 2009 Biotic and abiotic stress down-regulate miR398 expression in Arabidopsis. Planta 229, 1009–1014. 19148671 10.1007/s00425-009-0889-3

[CIT0116] Jagadish SV, Bahuguna RN, Djanaguiraman M, Gamuyao R, Prasad PV, Craufurd PQ 2016 Implications of high temperature and elevated CO_2_ on flowering time in plants. Frontiers in Plant Science 7, 913. 27446143 10.3389/fpls.2016.00913 PMC4921480

[CIT0117] Jagadish SVK, Craufurd PQ, Wheeler TR 2008 Phenotyping parents of mapping populations of rice for heat tolerance during anthesis. Crop Science 48, 1140–1146.

[CIT0118] Jagadish SVK, Muthurajan R, Oane R, Wheeler TR, Heuer S, Bennett J, Craufurd PQ 2010 Physiological and proteomic approaches to address heat tolerance during anthesis in rice (*Oryza sativa* L.). Journal of Experimental Botany 61, 143–156. 19858118 10.1093/jxb/erp289 PMC2791117

[CIT0119] Jaganathan D, Ramasamy K, Sellamuthu G, Jayabalan S, Venkataraman G 2018 CRISPR for crop improvement: an update review. Frontiers in Plant Science 9, 985. 30065734 10.3389/fpls.2018.00985 PMC6056666

[CIT0120] Jangid KK, Dwivedi P 2016 Physiological responses of drought stress in tomato: a review. International Journal of Agriculture, Environment and Biotechnology 9, 53.

[CIT0121] Janni M, Cadonici S, Bonas U, Grasso A, Dahab AAD, Visioli G, Pignone D, Ceriotti A, Marmiroli N 2018 Gene-ecology of durum wheat HMW glutenin reflects their diffusion from the center of origin. Scientific Reports 8, 16929. 30446715 10.1038/s41598-018-35251-4 PMC6240061

[CIT0122] Jessen D, Roth C, Wiermer M, Fulda M 2015 Two activities of long-chain acyl-coenzyme A synthetase are involved in lipid trafficking between the endoplasmic reticulum and the plastid in Arabidopsis. Plant Physiology 167, 351–366. 25540329 10.1104/pp.114.250365 PMC4326746

[CIT0123] Jha UC, Bohra A, Singh NP 2014 Heat stress in crop plants: its nature, impacts and integrated breeding strategies to improve heat tolerance. Plant Breeding 133, 679–701.

[CIT0124] Jiang J, Liu X, Liu C, Liu G, Li S, Wang L 2017 Integrating omics and alternative splicing reveals insights into grape response to high temperature. Plant Physiology 173, 1502–1518. 28049741 10.1104/pp.16.01305 PMC5291026

[CIT0125] Jin GH, Gho HJ, Jung KH 2013 A systematic view of rice heat shock transcription factor family using phylogenomic analysis. Journal of Plant Physiology 170, 321–329. 23122336 10.1016/j.jplph.2012.09.008

[CIT0126] Johnson SM, Lim F-L, Finkler A, Fromm H, Slabas AR, Knight MR 2014 Transcriptomic analysis of *Sorghum bicolor* responding to combined heat and drought stress. BMC Genomics 15, 456. 24916767 10.1186/1471-2164-15-456 PMC4070570

[CIT0127] Jones MO, Piron-Prunier F, Marcel F, et al. 2012 Characterisation of alleles of tomato light signalling genes generated by TILLING. Phytochemistry 79, 78–86. 22595361 10.1016/j.phytochem.2012.04.005

[CIT0128] Kaashyap M, Ford R, Bohra A, Kuvalekar A, Mantri N 2017 Improving salt tolerance of chickpea using modern genomics tools and molecular breeding. Current Genomics 18, 557–567. 29204084 10.2174/1389202918666170705155252 PMC5684649

[CIT0129] Kang NJ, Kang YI, Kang KH, Jeong BR 2009 Induction of thermotolerance and activation of antioxidant enzymes in H_2_O_2_ pre-applied leaves of cucumber and tomato seedlings. Journal of the Japanese Society for Horticultural Science 78, 320–329.

[CIT0130] Katiyar-Agarwal S, Agarwal M, Grover A 2003 Heat-tolerant basmati rice engineered by over-expression of hsp101. Plant Molecular Biology 51, 677–686. 12678556 10.1023/a:1022561926676

[CIT0131] Kaushal N, Bhandari K, Siddique KHM, Nayyar H 2016 Food crops face rising temperatures: an overview of responses, adaptive mechanisms, and approaches to improve heat tolerance. Cogent Food & Agriculture 2, 1134380.

[CIT0132] Keller M, Simm S; SPOT-ITN Consortium 2018 The coupling of transcriptome and proteome adaptation during development and heat stress response of tomato pollen. BMC Genomics 19, 447. 29884134 10.1186/s12864-018-4824-5 PMC5994098

[CIT0133] Key JL, Lin CY, Chen YM 1981 Heat shock proteins of higher plants. Proceedings of the National Academy of Sciences, USA 78, 3526–3530. 10.1073/pnas.78.6.3526 PMC319602 16593032

[CIT0134] Kilasi NL, Singh J, Vallejos CE, Ye C, Jagadish SVK, Kusolwa P, Rathinasabapathi B 2018 Heat stress tolerance in rice (*Oryza sativa* L.): identification of quantitative trait loci and candidate genes for seedling growth under heat stress. Frontiers in Plant Science 9, 1578. 30443261 10.3389/fpls.2018.01578 PMC6221968

[CIT0135] Kim D, Alptekin B, Budak H 2018 CRISPR/Cas9 genome editing in wheat. Functional & Integrative Genomics 18, 31–41. 28918562 10.1007/s10142-017-0572-x

[CIT0136] Kim M, Kim H, Lee W, Lee Y, Kwon S-W, Lee J 2015 Quantitative shotgun proteomics analysis of rice anther proteins after exposure to high temperature. International Journal of Genomics 2015, 1–9. 10.1155/2015/238704 PMC4651674 26618163

[CIT0137] Knoll JE, Ramos ML, Zeng Y, Holbrook CC, Chow M, Chen S, Maleki S, Bhattacharya A, Ozias-Akins P 2011 TILLING for allergen reduction and improvement of quality traits in peanut (*Arachis hypogaea* L.). BMC Plant Biology 11, 81. 21569438 10.1186/1471-2229-11-81 PMC3113929

[CIT0138] Koscielny CB, Gardner SW, Duncan RW 2018 Impact of high temperature on heterosis and general combining ability in spring canola (*Brassica napus* L.). Field Crops Research 221, 61–70.

[CIT0139] Kotak S, Larkindale J, Lee U, von Koskull-Döring P, Vierling E, Scharf KD 2007 Complexity of the heat stress response in plants. Current Opinion in Plant Biology 10, 310–316. 17482504 10.1016/j.pbi.2007.04.011

[CIT0140] Krasileva KV, Vasquez-Gross HA, Howell T, et al. 2017 Uncovering hidden variation in polyploid wheat. Proceedings of the National Academy of Sciences, USA 114, E913–E921. 10.1073/pnas.1619268114 PMC5307431 28096351

[CIT0141] Krishnamurthy L, Gaur PM, Basu PS, Chaturvedi SK, Tripathi S, Vadez V, Rathore A, Varshney RK, Gowda CLL 2011 Large genetic variation for heat tolerance in the reference collection of chickpea (*Cicer arietinum* L.) germplasm. Plant Genetic Resources 9, 59–69.

[CIT0142] Krishnan A, Gupta V, Ritvik, Nongkynrih B, Thakur J 2011 How to effectively monitor and evaluate NCD programmes in India. Indian Journal of Community Medicine 36, S57–S62. 22628913 10.4103/0970-0218.94710 PMC3354904

[CIT0143] Kumar AP, Boualem A, Bhattacharya A, Parikh S, Desai N, Zambelli A, Leon A, Chatterjee M, Bendahmane A 2013 SMART—Sunflower Mutant population And Reverse genetic Tool for crop improvement. BMC Plant Biology 13, 38. 23496999 10.1186/1471-2229-13-38 PMC3606330

[CIT0144] Kumar APK, McKeown PC, Boualem A, Ryder P, Brychkova G, Bendahmane A, Sarkar A, Chatterjee M, Spillane C 2017 TILLING by sequencing (TbyS) for targeted genome mutagenesis in crops. Molecular Breeding 37, 14.

[CIT0145] Kumar RR, Pathak H, Sharma SK, Kala YK, Nirjal MK, Singh GP, Goswami S, Rai RD 2015 Novel and conserved heat-responsive microRNAs in wheat (*Triticum aestivum* L.). Functional & Integrative Genomics 15, 323–348. 25480755 10.1007/s10142-014-0421-0

[CIT0146] Kumar RR, Singh K, Ahuja S, et al. 2019 Quantitative proteomic analysis reveals novel stress-associated active proteins (SAAPs) and pathways involved in modulating tolerance of wheat under terminal heat. Functional & Integrative Genomics 19, 329–348. 30465139 10.1007/s10142-018-0648-2

[CIT0147] Lamaoui M, Jemo M, Datla R, Bekkaoui F 2018 Heat and drought stresses in crops and approaches for their mitigation. Frontiers in Chemistry 6, 26. 29520357 10.3389/fchem.2018.00026 PMC5827537

[CIT0148] Lavania D, Dhingra A, Grover A 2018 Analysis of transactivation potential of rice (*Oryza sativa* L.) heat shock factors. Planta 247, 1267–1276. 29453664 10.1007/s00425-018-2865-2

[CIT0149] Li M, Li X, Zhou Z, Wu P, Fang M, Pan X, Lin Q, Luo W, Wu G, Li H 2016 Reassessment of the four yield-related genes Gn1a, DEP1, GS3, and IPA1 in rice using a CRISPR/Cas9 system. Frontiers in Plant Science 7, 377. 27066031 10.3389/fpls.2016.00377 PMC4811884

[CIT0150] Li N, Gügel IL, Giavalisco P, Zeisler V, Schreiber L, Soll J, Philippar K 2015 FAX1, a novel membrane protein mediating plastid fatty acid export. PLoS Biology 13, e1002053. 25646734 10.1371/journal.pbio.1002053 PMC4344464

[CIT0151] Li X, Lawas LM, Malo R, et al. 2015 Metabolic and transcriptomic signatures of rice floral organs reveal sugar starvation as a factor in reproductive failure under heat and drought stress. Plant, Cell & Environment 38, 2171–2192. 10.1111/pce.12545 25828772

[CIT0152] Li XM, Chao DY, Wu Y, et al. 2015 Natural alleles of a proteasome α2 subunit gene contribute to thermotolerance and adaptation of African rice. Nature Genetics 47, 827–833. 25985140 10.1038/ng.3305

[CIT0153] Li Y, Yu Z, Jin J, Zhang Q, Wang G, Liu C, Wu J, Wang C, Liu X 2018 Impact of elevated CO_2_ on seed quality of soybean at the fresh edible and mature stages. Frontiers in Plant Science 9, 1413. 30386351 10.3389/fpls.2018.01413 PMC6199416

[CIT0154] Liao J-L, Zhou H-W, Peng Q, Zhong P-A, Zhang H-Y, He C, Huang Y-J 2015 Transcriptome changes in rice (*Oryza sativa* L.) in response to high night temperature stress at the early milky stage. BMC Genomics 16, 18. 25928563 10.1186/s12864-015-1222-0 PMC4369907

[CIT0155] Liao J-L, Zhou H-W, Zhang H-Y, Zhong P-A, Huang Y-J 2014 Comparative proteomic analysis of differentially expressed proteins in the early milky stage of rice grains during high temperature stress. Journal of Experimental Botany 65, 655–671. 24376254 10.1093/jxb/ert435 PMC3904723

[CIT0156] Lim GH, Singhal R, Kachroo A, Kachroo P 2017 Fatty acid- and lipid-mediated signaling in plant defense. Annual Review of Phytopathology 55, 505–536. 10.1146/annurev-phyto-080516-035406 28777926

[CIT0157] Lin H-H, Lin K-H, Syu J-Y, Tang S-Y, Lo H-F 2016 Physiological and proteomic analysis in two wild tomato lines under waterlogging and high temperature stress. Journal of Plant Biochemistry and Biotechnology 25, 87–96.

[CIT0158] Lin YX, Jiang HY, Chu ZX, Tang XL, Zhu SW, Cheng BJ 2011 Genome-wide identification, classification and analysis of heat shock transcription factor family in maize. BMC Genomics 12, 76. 21272351 10.1186/1471-2164-12-76 PMC3039612

[CIT0159] Ling Y, Serrano N, Gao G, et al. 2018 Thermopriming triggers splicing memory in Arabidopsis. Journal of Experimental Botany 69, 2659–2675. 29474581 10.1093/jxb/ery062 PMC5920379

[CIT0160] Liu C-W, Chang T-S, Hsu Y-K, Wang AZ, Yen H-C, Wu Y-P, Wang C-S, Lai C-C 2014 Comparative proteomic analysis of early salt stress responsive proteins in roots and leaves of rice. Proteomics 14, 1759–1775. 24841874 10.1002/pmic.201300276

[CIT0161] Liu GT, Wang JF, Cramer G, Dai ZW, Duan W, Xu HG, Wu BH, Fan PG, Wang LJ, Li SH 2012 *a* Transcriptomic analysis of grape (*Vitis vinifera* L.) leaves during and after recovery from heat stress. BMC Plant Biology 12, 174. 23016701 10.1186/1471-2229-12-174 PMC3497578

[CIT0162] Liu GT, Wang JF, Cramer G, Dai ZW, Duan W, Xu HG, Wu BH, Fan PG, Wang LJ, Li SH 2012 *b* Transcriptomic analysis of grape (*Vitis vinifera* L.) leaves during and after recovery from heat stress. BMC Plant Biology 12, 174. 23016701 10.1186/1471-2229-12-174 PMC3497578

[CIT0163] Liu Q, Yan S, Yang T, Zhang S, Chen YQ, Liu B 2017 Small RNAs in regulating temperature stress response in plants. Journal of Integrative Plant Biology 59, 774–791. 28731217 10.1111/jipb.12571

[CIT0164] Liu X, Huang B 2000 Heat stress injury in relation to membrane lipid peroxidation in creeping bentgrass. Crop Science 40, 503–510.

[CIT0165] Liu Y, Li J, Zhu Y, Jones A, Rose RJ, Song Y 2019 Heat stress in legume seed setting: effects, causes, and future prospects. Frontiers in Plant Science 10, 938. 31417579 10.3389/fpls.2019.00938 PMC6684746

[CIT0166] Liu Z, Xin M, Qin J, Peng H, Ni Z, Yao Y, Sun Q 2015 Temporal transcriptome profiling reveals expression partitioning of homeologous genes contributing to heat and drought acclimation in wheat (*Triticum aestivum* L.). BMC Plant Biology 15, 152. 26092253 10.1186/s12870-015-0511-8 PMC4474349

[CIT0167] Lobell DB, Schlenker W, Costa-Roberts J 2011 Climate trends and global crop production since 1980. Science 333, 616–620. 21551030 10.1126/science.1204531

[CIT0168] Lopes MS, El-Basyoni I, Baenziger PS, et al. 2015 Exploiting genetic diversity from landraces in wheat breeding for adaptation to climate change. Journal of Experimental Botany 66, 3477–3486. 25821073 10.1093/jxb/erv122

[CIT0169] Lorenz R, Stalhandske Z, Fischer EM 2019 Detection of a climate change signal in extreme heat, heat stress, and cold in europe from observations. Geophysical Research Letters 46, 8363–8374.

[CIT0170] Lu Y, Li R, Wang R, Wang X, Zheng W, Sun Q, Tong S, Dai S, Xu S 2017 Comparative proteomic analysis of flag leaves reveals new insight into wheat heat adaptation. Frontiers in Plant Science 8, 1086. 28676819 10.3389/fpls.2017.01086 PMC5476934

[CIT0171] Lucas MR, Ehlers JD, Huynh B-L, Diop N-N, Roberts PA, Close TJ 2013 Markers for breeding heat-tolerant cowpea. Molecular Breeding 31, 529–536.

[CIT0172] Luo Q 2011 Temperature thresholds and crop production: a review. Climate Change 109, 583–598.

[CIT0173] MacAlister CA, Park SJ, Jiang K, Marcel F, Bendahmane A, Izkovich Y, Eshed Y, Lippman ZB 2012 Synchronization of the flowering transition by the tomato *TERMINATING FLOWER* gene. Nature Genetics 44, 1393–1398. 23143603 10.1038/ng.2465

[CIT0174] Maestri E, Klueva N, Perrotta C, Gulli M, Nguyen HT, Marmiroli N 2002 Molecular genetics of heat tolerance and heat shock proteins in cereals. Plant Molecular Biology 48, 667–681. 11999842 10.1023/a:1014826730024

[CIT0175] Makarova S, Makhotenko A, Spechenkova N, Love AJ, Kalinina NO, Taliansky M 2018 Interactive responses of potato (*Solanum tuberosum* L.) plants to heat stress and infection with Potato virus Y. Frontiers in Microbiology 9, 2582. 30425697 10.3389/fmicb.2018.02582 PMC6218853

[CIT0176] Malcevschi A, Marmiroli N 2012 Plant protein analysis. In: Hazelwood J, ed. Proteomic applications in biology. IntechOpen.

[CIT0177] Mangelsen E, Kilian J, Harter K, Jansson C, Wanke D, Sundberg E 2011 Transcriptome analysis of high-temperature stress in developing barley caryopses: early stress responses and effects on storage compound biosynthesis. Molecular Plant 4, 97–115. 20924027 10.1093/mp/ssq058

[CIT0178] Mangrauthia SK, Agarwal S, Sailaja B, Sarla N, Voleti SR 2016 Transcriptome analysis of *Oryza sativa* (rice) seed germination at high temperature shows dynamics of genome expression associated with hormones signalling and abiotic stress pathways. Tropical Plant Biology 9, 215–228.

[CIT0179] Mangrauthia SK, Bhogireddy S, Agarwal S, Prasanth VV, Voleti SR, Neelamraju S, Subrahmanyam D 2017 Genome-wide changes in microRNA expression during short and prolonged heat stress and recovery in contrasting rice cultivars. Journal of Experimental Botany 68, 2399–2412. 28407080 10.1093/jxb/erx111 PMC5447883

[CIT0180] Marfo KO, Hall AE 1992 Inheritance of heat tolerance during pod set in Cowpea. Crop Science 32, 912.

[CIT0181] Marko D, El-shershaby A, Carriero F, Summerer S, Petrozza A, Iannacone R, Schleiff E, Fragkostefanakis S 2019 Identification and characterization of a thermotolerant TILLING allele of heat shock binding protein 1 in tomato. Genes 10, 516. 10.3390/genes10070516 PMC6678839 31284688

[CIT0182] Martinière A, Shvedunova M, Thomson AJ, Evans NH, Penfield S, Runions J, McWatters HG 2011 Homeostasis of plasma membrane viscosity in fluctuating temperatures. New Phytologist 192, 328–337. 21762166 10.1111/j.1469-8137.2011.03821.x

[CIT0183] Matei G, Woyann LG, Milioli AS, de Bem Oliveira I, Zdziarski AD, Zanella R, Coelho ASG, Finatto T, Benin G 2018 Genomic selection in soybean: accuracy and time gain in relation to phenotypic selection. Molecular Breeding 38, 117.

[CIT0184] Maulana F, Ayalew H, Anderson JD, Kumssa TT, Huang W, Ma XF 2018 Genome-wide association mapping of seedling heat tolerance in winter wheat. Frontiers in Plant Science 9, 1272. 30233617 10.3389/fpls.2018.01272 PMC6131858

[CIT0185] Mazzeo MF, Cacace G, Iovieno P, Massarelli I, Grillo S, Siciliano RA 2018 Response mechanisms induced by exposure to high temperature in anthers from thermo-tolerant and thermo-sensitive tomato plants: a proteomic perspective. PLoS One 13, e0201027. 30024987 10.1371/journal.pone.0201027 PMC6053223

[CIT0186] Meseka S, Menkir A, Bossey B, Mengesha W 2018 Performance assessment of drought tolerant maize hybrids under combined drought and heat stress. Agronomy 8, 274. 10.3390/agronomy8120274 PMC7672364 33304638

[CIT0187] Miller MJ, Vierstra RD 2011 Mass spectrometric identification of SUMO substrates provides insights into heat stress-induced SUMOylation in plants. Plant Signaling & Behavior 6, 130–133. 21270536 10.4161/psb.6.1.14256 PMC3122025

[CIT0188] Mittal D, Chakrabarti S, Sarkar A, Singh A, Grover A 2009 Heat shock factor gene family in rice: genomic organization and transcript expression profiling in response to high temperature, low temperature and oxidative stresses. Plant Physiology and Biochemistry 47, 785–795. 19539489 10.1016/j.plaphy.2009.05.003

[CIT0189] Mittal D, Madhyastha DA, Grover A 2012 Gene expression analysis in response to low and high temperature and oxidative stresses in rice: combination of stresses evokes different transcriptional changes as against stresses applied individually. Plant Science 197, 102–113. 23116677 10.1016/j.plantsci.2012.09.008

[CIT0190] Mobin M, Khan MN, Abbas ZK, Ansari HR, Al-Mutairi KA 2017 Significance of sulfur in heat stressed cluster bean (*Cymopsis tetragonoloba* L. Taub) genotypes: responses of growth, sugar and antioxidative metabolism. Archives of Agronomy and Soil Science 63, 288–295.

[CIT0191] Montero-Barrientos M, Hermosa R, Nicolás C, Cardoza RE, Gutiérrez S, Monte E 2008 Overexpression of a *Trichoderma* HSP70 gene increases fungal resistance to heat and other abiotic stresses. Fungal Genetics and Biology 45, 1506–1513. 18824239 10.1016/j.fgb.2008.09.003

[CIT0192] Mu Q, Zhang W, Zhang Y, Yan H, Liu K, Matsui T, Tian X, Yang P 2017 iTRAQ-based quantitative proteomics analysis on rice anther responding to high temperature. International Journal of Molecular Sciences 18, 1811. 10.3390/ijms18091811 PMC5618475 28832496

[CIT0193] Mueller SP, Unger M, Guender L, Fekete A, Mueller MJ 2017 Phospholipid:diacylglycerol acyltransferase-mediated triacylglyerol synthesis augments basal thermotolerance. Plant Physiology 175, 486–497. 28733391 10.1104/pp.17.00861 PMC5580778

[CIT0194] Muhlemann JK, Younts TLB, Muday GK 2018 Flavonols control pollen tube growth and integrity by regulating ROS homeostasis during high-temperature stress. Proceedings of the National Academy of Sciences, USA 115, E11188–E11197. 10.1073/pnas.1811492115 PMC6255205 30413622

[CIT0195] Muth J, Hartje S, Twyman RM, Hofferbert H-R, Tacke E, Prüfer D 2008 Precision breeding for novel starch variants in potato. Plant Biotechnology Journal 6, 576–584. 18422889 10.1111/j.1467-7652.2008.00340.x

[CIT0196] Nahar K, Hasanuzzaman M, Alam MdM, Fujita M 2015 Exogenous glutathione confers high temperature stress tolerance in mung bean (*Vigna radiata* L.) by modulating antioxidant defense and methylglyoxal detoxification system. Environmental and Experimental Botany 112, 44–54.

[CIT0197] Narayanan S, Prasad PV, Welti R 2016 Wheat leaf lipids during heat stress: II. Lipids experiencing coordinated metabolism are detected by analysis of lipid co-occurrence. Plant, Cell & Environment 39, 608–617. 10.1111/pce.12648 PMC5141584 26436445

[CIT0198] Narayanan S, Prasad PVV, Welti R 2018 Alterations in wheat pollen lipidome during high day and night temperature stress. Plant, Cell & Environment 41, 1749–1761. 10.1111/pce.13156 PMC6713575 29377219

[CIT0199] Navarro-Reig M, Tauler R, Iriondo-Frias G, Jaumot J 2019 Untargeted lipidomic evaluation of hydric and heat stresses on rice growth. Journal of Chromatography. B, Analytical Technologies in the Biomedical and Life Sciences 1104, 148–156. 30471516 10.1016/j.jchromb.2018.11.018

[CIT0200] Naveed M, Ahsan M, Akram HM, Aslam M, Ahmed N 2016 Genetic effects conferring heat tolerance in a cross of tolerant × susceptible maize (*Zea mays* L.) Genotypes. Frontiers in Plant Science 7, 729. 27313583 10.3389/fpls.2016.00729 PMC4889604

[CIT0201] Nelson DE, Repetti PP, Adams TR, et al. 2007 Plant nuclear factor Y (NF-Y) B subunits confer drought tolerance and lead to improved corn yields on water-limited acres. Proceedings of the National Academy of Sciences, USA 104, 16450–16455. 10.1073/pnas.0707193104 PMC2034233 17923671

[CIT0202] Neta-Sharir I, Isaacson T, Lurie S, Weiss D 2005 Dual role for tomato heat shock protein 21: protecting photosystem II from oxidative stress and promoting color changes during fruit maturation. The Plant Cell 17, 1829–1838. 15879560 10.1105/tpc.105.031914 PMC1143080

[CIT0203] Ni Z, Li H, Zhao Y, Peng H, Hu Z, Xin M, Sun Q 2018 Genetic improvement of heat tolerance in wheat: recent progress in understanding the underlying molecular mechanisms. The Crop Journal 6, 32–41.

[CIT0204] Niu Y, Xiang Y 2018 An overview of biomembrane functions in plant responses to high-temperature stress. Frontiers in Plant Science 9, 915. 30018629 10.3389/fpls.2018.00915 PMC6037897

[CIT0205] Ohama N, Sato H, Shinozaki K, Yamaguchi-Shinozaki K 2017 Transcriptional regulatory network of plant heat stress response. Trends in Plant Science 22, 53–65. 27666516 10.1016/j.tplants.2016.08.015

[CIT0206] Okabe Y, Ariizumi T, Ezura H 2013 Updating the Micro-Tom TILLING platform. Breeding Science 63, 42–48. 23641180 10.1270/jsbbs.63.42 PMC3621444

[CIT0207] Okabe Y, Asamizu E, Saito T, Matsukura C, Ariizumi T, Brès C, Rothan C, Mizoguchi T, Ezura H 2011 Tomato TILLING technology: development of a reverse genetics tool for the efficient isolation of mutants from micro-Tom mutant libraries. Plant & Cell Physiology 52, 1994–2005. 21965606 10.1093/pcp/pcr134 PMC3212723

[CIT0208] Osmond CB, Austin MP, Berry JA, Billings WD, Boyer JS, Dacey JWH, Nobel PS, Smith SD, Winner WE 1987 Stress physiology and the distribution of plants. BioScience 37, 38–48.

[CIT0209] Otero A, Goni C, Jifon JL, Syvertsen JP 2011 High temperature effects on citrus orange leaf gas exchange, flowering, fruit quality and yield. Acta Horticulturae 1069–1075.

[CIT0210] Padi FK, Denwar NN, Kaleem FZ, Salifu AB, Clottey VA, Kombiok J, Haruna M, Hall AE, Marfo KO 2004 Registration of ‘Apagbaala’ cowpea. Crop Science 44, 1486.

[CIT0211] Paliwal R, Röder MS, Kumar U, Srivastava JP, Joshi AK 2012 QTL mapping of terminal heat tolerance in hexaploid wheat (*T. aestivum* L.). Theoretical and Applied Genetics 125, 561–575. 22476874 10.1007/s00122-012-1853-3

[CIT0212] Parankusam S, Bhatnagar-Mathur P, Sharma KK 2017 Heat responsive proteome changes reveal molecular mechanisms underlying heat tolerance in chickpea. Environmental and Experimental Botany 141, 132–144.

[CIT0213] Park CJ, Seo YS 2015 Heat shock proteins: a review of the molecular chaperones for plant immunity. Plant Pathology Journal 31, 323–333. 26676169 10.5423/PPJ.RW.08.2015.0150 PMC4677741

[CIT0214] Paupière MJ, van Haperen P, Rieu I, Visser RGF, Tikunov YM, Bovy AG 2017 Screening for pollen tolerance to high temperatures in tomato. Euphytica 213, 130.

[CIT0215] Petrov VD, Van Breusegem F 2012 Hydrogen peroxide—a central hub for information flow in plant cells. AoB Plants 2012, pls014. 22708052 10.1093/aobpla/pls014 PMC3366437

[CIT0216] Phan TTT, Ishibashi Y, Miyazaki M, Tran HT, Okamura K, Tanaka S, Nakamura J, Yuasa T, Iwaya-Inoue M 2013 High temperature-induced repression of the rice sucrose transporter (OsSUT1) and starch synthesis-related genes in sink and source organs at milky ripening stage causes chalky grains. Journal of Agronomy and Crop Scence 199, 178–188.

[CIT0217] Pignone D, De Paola D, Rapanà N, Janni M 2015 Single seed descent: a tool to exploit durum wheat (*Triticum durum* Desf.) genetic resources. Genetic Resources and Crop Evolution 62, 1029–1035.

[CIT0218] Piron F, Nicolaï M, Minoïa S, Piednoir E, Moretti A, Salgues A, Zamir D, Caranta C, Bendahmane A 2010 An induced mutation in tomato eIF4E leads to immunity to two potyviruses. PLoS One 5, e11313. 20593023 10.1371/journal.pone.0011313 PMC2892489

[CIT0219] Polanía JA, Chaves N, Lobaton JD, Cajiao VCH, Rao IM, Raatz B, Beebe SE 2017 Heat tolerance in common bean derived from interspecific crosses. International Center for Tropical Agriculture (CIAT) https://hdl.handle.net/10568/89450

[CIT0220] Pottorff M, Roberts PA, Close TJ, Lonardi S, Wanamaker S, Ehlers JD 2014 Identification of candidate genes and molecular markers for heat-induced brown discoloration of seed coats in cowpea [*Vigna unguiculata* (L.) Walp]. BMC Genomics 15, 328. 24885083 10.1186/1471-2164-15-328 PMC4035059

[CIT0221] Prasad PVV, Bheemanahalli R, Jagadish SVK 2017 Field crops and the fear of heat stress—opportunities, challenges and future directions. Field Crops Research 200, 114–121.

[CIT0222] Prasad PVV, Craufurd PQ, Kakani VG, Wheeler TR, Boote KJ 2001 Influence of high temperature during pre- and post-anthesis stages of floral development on fruit-set and pollen germination in peanut. Functional Plant Biology 28, 233–240.

[CIT0223] Prasad PVV, Djanaguiraman M 2014 Response of floret fertility and individual grain weight of wheat to high temperature stress: sensitive stages and thresholds for temperature and duration. Functional Plant Biology 41, 1261–1269. 32481075 10.1071/FP14061

[CIT0224] Prasad PVV, Pisipati SR, Mutava RN, Tuinstra MR 2008 Sensitivity of grain sorghum to high temperature stress during reproductive development. Crop Science 48, 1911.

[CIT0225] Priya M, Dhanker OP, Siddique KHM, HanumanthaRao B, Nair RM, Pandey S, Singh S, Varshney RK, Prasad PVV, Nayyar H 2019 Drought and heat stress-related proteins: an update about their functional relevance in imparting stress tolerance in agricultural crops. Theoretical and Applied Genetics 132, 1607–1638. 30941464 10.1007/s00122-019-03331-2

[CIT0226] Prasad R, Gunn SK, Rotz CA, Karsten H, Roth G, Buda A, Stoner AMK 2018 Projected climate and agronomic implications for corn production in the Northeastern United States. PLoS One 13, e0198623. 29889853 10.1371/journal.pone.0198623 PMC5995377

[CIT0227] Qi Y, Wang H, Zou Y, Liu C, Liu Y, Wang Y, Zhang W 2011 Over-expression of mitochondrial heat shock protein 70 suppresses programmed cell death in rice. FEBS Letters 585, 231–239. 21130768 10.1016/j.febslet.2010.11.051

[CIT0227a] Qin D, Wang F, Geng X, Zhang L, Yao Y, Ni Z, Peng H, Sun Q 2015 Overexpression of heat stress-responsive TaMBF1c, a wheat (Triticum aestivum L.) Multiprotein Bridging Factor, confers heat tolerance in both yeast and rice. Plant Molecular Biology 87, 31–45. 10.1007/s11103-014-0259-9 25326264

[CIT0228] Qin D, Wu H, Peng H, Yao Y, Ni Z, Li Z, Zhou C, Sun Q 2008 Heat stress-responsive transcriptome analysis in heat susceptible and tolerant wheat (*Triticum aestivum* L.) by using wheat genome array. BMC Genomics 9, 432. 18808683 10.1186/1471-2164-9-432 PMC2614437

[CIT0229] Qu AL, Ding YF, Jiang Q, Zhu C 2013 Molecular mechanisms of the plant heat stress response. Biochemical and Biophysical Research Communications 432, 203–207. 23395681 10.1016/j.bbrc.2013.01.104

[CIT0230] Qu M, Chen G, Bunce JA, Zhu X, Sicher RC 2018 Systematic biology analysis on photosynthetic carbon metabolism of maize leaf following sudden heat shock under elevated CO_2_. Scientific Reports 8, 7849. 29777170 10.1038/s41598-018-26283-x PMC5959914

[CIT0231] Rahaman M, Mamidi S, Rahman M 2018 Genome-wide association study of heat stress-tolerance traits in spring-type *Brassica napus* L. under controlled conditions. The Crop Journal 6, 115–125.

[CIT0232] Ravichandran S, Ragupathy R, Edwards T, Domaratzki M, Cloutier S 2019 MicroRNA-guided regulation of heat stress response in wheat. BMC Genomics 20, 488. 31195958 10.1186/s12864-019-5799-6 PMC6567507

[CIT0233] Raza A, Razzaq A, Mehmood S, Zou X, Zhang X, Lv Y, Xu J 2019 Impact of climate change on crops adaptation and strategies to tackle its outcome: a review. Plants 8, 34. 10.3390/plants8020034 PMC6409995 30704089

[CIT0234] Reddy TV, Dwivedi S, Sharma NK 2012 Development of TILLING by sequencing platform towards enhanced leaf yield in tobacco. Industrial Crops and Products 40, 324–335.

[CIT0235] Reynolds M, Bonnett D, Chapman SC, Furbank RT, Manès Y, Mather DE, Parry MA 2011 Raising yield potential of wheat. I. Overview of a consortium approach and breeding strategies. Journal of Experimental Botany 62, 439–452. 20952629 10.1093/jxb/erq311

[CIT0236] Ricroch A, Clairand P, Harwood W 2017 Use of CRISPR systems in plant genome editing: toward new opportunities in agriculture. Emerging Topics in Life Sciences 1, 169–182. 10.1042/ETLS20170085 PMC7288993 33525765

[CIT0237] Roorkiwal M, Rathore A, Das RR, et al. 2016 Genome-enabled prediction models for yield related traits in chickpea. Frontiers in Plant Science 7, 1666. 27920780 10.3389/fpls.2016.01666 PMC5118446

[CIT0238] Ruggieri V, Calafiore R, Schettini C, Rigano M, Olivieri F, Frusciante L, Barone A 2019 Exploiting genetic and genomic resources to enhance heat-tolerance in tomatoes. Agronomy 9, 22.

[CIT0239] Salvi P, Kamble NU, Majee M 2018 Stress-inducible galactinol synthase of chickpea (CaGolS) is implicated in heat and oxidative stress tolerance through reducing stress-induced excessive reactive oxygen species accumulation. Plant & Cell Physiology 59, 155–166. 29121266 10.1093/pcp/pcx170

[CIT0240] Sanyal RP, Misra HS, Saini A 2018 Heat-stress priming and alternative splicing-linked memory. Journal of Experimental Botany 69, 2431–2434. 29718462 10.1093/jxb/ery111 PMC5920290

[CIT0241] Sarkar NK, Kim YK, Grover A 2014 Coexpression network analysis associated with call of rice seedlings for encountering heat stress. Plant Molecular Biology 84, 125–143. 23975147 10.1007/s11103-013-0123-3

[CIT0242] Scharf KD, Berberich T, Ebersberger I, Nover L 2012 The plant heat stress transcription factor (Hsf) family: structure, function and evolution. Biochimica et Biophysica Acta 1819, 104–119. 22033015 10.1016/j.bbagrm.2011.10.002

[CIT0243] Scott JW, Olson SM, Howe TK, Stoffella PJ, Bartz JA, Bryan HH 1995 ‘Equinox’ heat-tolerant hybrid tomato. HortScience 30, 647–648.

[CIT0244] Sedeek KEM, Mahas A, Mahfouz M 2019 Plant genome engineering for targeted improvement of crop traits. Frontiers in Plant Science 10, 114. 30809237 10.3389/fpls.2019.00114 PMC6379297

[CIT0245] Sehgal A, Sita K, Kumar J, Kumar S, Singh S, Siddique KHM, Nayyar H 2017 Effects of drought, heat and their interaction on the growth, yield and photosynthetic function of lentil (*Lens culinaris* Medikus) genotypes varying in heat and drought sensitivity. Frontiers in Plant Science 8, 1776. 29089954 10.3389/fpls.2017.01776 PMC5651046

[CIT0246] Sestili F, Palombieri S, Botticella E, Mantovani P, Bovina R, Lafiandra D 2015 TILLING mutants of durum wheat result in a high amylose phenotype and provide information on alternative splicing mechanisms. Plant Science 233, 127–133. 25711820 10.1016/j.plantsci.2015.01.009

[CIT0247] Setia RC, Setia N 2008 The ‘-OMICS’ technologies and crop improvement. In: Setia RC, Nayyar H, Setia N, eds. Crop improvement:strategies and applications. New Dehli: International Publishing House Pvt. Ltd, 1–18.

[CIT0248] Shah F, Huang J, Cui K, Nie L, Shah T, Chen C, Wang K 2011 Impact of high-temperature stress on rice plant and its traits related to tolerance. Journal of Agricultural Science 149, 545–556.

[CIT0249] Shan Q, Wang Y, Li J, et al. 2013 Targeted genome modification of crop plants using a CRISPR–Cas system. Nature Biotechnology 31, 686–688. 10.1038/nbt.2650 23929338

[CIT0250] Shan Q, Wang Y, Li J, Gao C 2014 Genome editing in rice and wheat using the CRISPR/Cas system. Nature Protocols 9, 2395–2410. 25232936 10.1038/nprot.2014.157

[CIT0251] Shanmugavadivel PS, Amitha Mithra SV, Prakash C, MK R, Tiwari R, Mohapatra T, Singh NK 2017 High resolution mapping of QTLs for heat tolerance in rice using a 5K SNP array. Rice 10, 28. 28584974 10.1186/s12284-017-0167-0 PMC5459777

[CIT0252] Sharma DK, Torp AM, Rosenqvist E, Ottosen CO, Andersen SB 2017 QTLs and potential candidate genes for heat stress tolerance identified from the mapping populations specifically segregating for Fv/Fm in wheat. Frontiers in Plant Science 8, 1668. 29021798 10.3389/fpls.2017.01668 PMC5623722

[CIT0253] Sharma L, Dalal M, Verma RK, Kumar SVV, Yadav SK, Pushkar S, Kushwaha SR, Bhowmik A, Chinnusamy V 2018 Auxin protects spikelet fertility and grain yield under drought and heat stresses in rice. Environmental and Experimental Botany 150, 9–24.

[CIT0254] Shen L, Hua Y, Fu Y, et al. 2017 Rapid generation of genetic diversity by multiplex CRISPR/Cas9 genome editing in rice. Science China Life Sciences 60, 506–515. 28349304 10.1007/s11427-017-9008-8

[CIT0255] Shi J, Gao H, Wang H, Lafitte HR, Archibald RL, Yang M, Hakimi SM, Mo H, Habben JE 2017 *a* ARGOS8 variants generated by CRISPR–Cas9 improve maize grain yield under field drought stress conditions. Plant Biotechnology Journal 15, 207–216. 27442592 10.1111/pbi.12603 PMC5258859

[CIT0256] Shi J, Yan B, Lou X, Ma H, Ruan S 2017 *b* Comparative transcriptome analysis reveals the transcriptional alterations in heat-resistant and heat-sensitive sweet maize (*Zea mays* L.) varieties under heat stress. BMC Plant Biology 17, 26. 28122503 10.1186/s12870-017-0973-y PMC5267381

[CIT0257] Shirdelmoghanloo H, Taylor JD, Lohraseb I, Rabie H, Brien C, Timmins A, Martin P, Mather DE, Emebiri L, Collins NC 2016 A QTL on the short arm of wheat (*Triticum aestivum* L.) chromosome 3B affects the stability of grain weight in plants exposed to a brief heat shock early in grain filling. BMC Plant Biology 16, 100. 27101979 10.1186/s12870-016-0784-6 PMC4841048

[CIT0258] Silletti MF, Petrozza A, Stigliani AL, Giorio G, Cellini F, D’Ambrosio C, Carriero F 2013 An increase of lycopene content in tomato fruit is associated with a novel Cyc-B allele isolated through TILLING technology. Molecular Breeding 31, 665–674.

[CIT0259] Singh D, Balota M, Collakova E, Isleib TG, Welbaum GE, Tallury SP 2016 Heat stress related physiological and metabolic traits in peanut seedlings. Peanut Science 43, 24–35.

[CIT0260] Singh S, Vikram P, Sehgal D, et al. 2018 Harnessing genetic potential of wheat germplasm banks through impact-oriented-prebreeding for future food and nutritional security. Scientific Reports 8, 12527. 30131572 10.1038/s41598-018-30667-4 PMC6104032

[CIT0261] Singh R, Jwa NS 2013 Understanding the responses of rice to environmental stress using proteomics. Journal of Proteome Research 12, 4652–4669. 23984864 10.1021/pr400689j

[CIT0262] Sita K, Sehgal A, Bhandari K, Kumar J, Kumar S, Singh S, Siddique KH, Nayyar H 2018 Impact of heat stress during seed filling on seed quality and seed yield in lentil (*Lens culinaris* Medikus) genotypes. Journal of the Science of Food and Agriculture 98, 5134–5141. 29635707 10.1002/jsfa.9054

[CIT0263] Slade AJ, McGuire C, Loeffler D, et al. 2012 Development of high amylose wheat through TILLING. BMC Plant Biology 12, 69. 22584013 10.1186/1471-2229-12-69 PMC3424102

[CIT0264] Snider JL, Russo VM, Roberts W, Wann EV, Raper RL 2012 Cultural and environmental factors governing tomato production: local production under elevated temperatures. HortScience 47, 1022–1028.

[CIT0265] Soda N, Wallace SA 2015 Omics study for abiotic stress responses in plants. Advances in Plants & Agricultural Research 2, 00037.

[CIT0266] Song J, Carver BF, Powers C, Yan L, Klápště J, El-Kassaby YA, Chen C 2017 Practical application of genomic selection in a doubled-haploid winter wheat breeding program. Molecular Breeding 37, 117. 28936114 10.1007/s11032-017-0715-8 PMC5582076

[CIT0267] Sparks DL 2018 Advances in agronomy. New York: Academic Press.

[CIT0268] Sparla F, Falini G, Botticella E, Pirone C, Talamè V, Bovina R, Salvi S, Tuberosa R, Sestili F, Trost P 2014 New starch phenotypes produced by TILLING in barley. PLoS One 9, e107779. 25271438 10.1371/journal.pone.0107779 PMC4182681

[CIT0269] Spicher L, Glauser G, Kessler F 2016 Lipid antioxidant and galactolipid remodeling under temperature stress in tomato plants. Frontiers in Plant Science 7, 167. 26925083 10.3389/fpls.2016.00167 PMC4756161

[CIT0270] Spindel J, Iwata H 2018 Genomic selection in rice breeding. In: Sasaki T, Ashikari M, eds. Rice genomics, genetics and breeding. Singapore: Springer Singapore, 473–496.

[CIT0271] Stief A, Altmann S, Hoffmann K, Pant BD, Scheible WR, Bäurle I 2014 Arabidopsis miR156 regulates tolerance to recurring environmental stress through SPL transcription factors. The Plant Cell 26, 1792–1807. 24769482 10.1105/tpc.114.123851 PMC4036586

[CIT0272] Sung D-Y, Kaplan F, Guy CL 2001 Plant Hsp70 molecular chaperones: protein structure, gene family, expression and function. Physiologia Plantarum 113, 443–451.

[CIT0273] Supek F, Bošnjak M, Škunca N, Šmuc T 2011 REVIGO summarizes and visualizes long lists of Gene Ontology terms. PLoS One 6, e21800. 21789182 10.1371/journal.pone.0021800 PMC3138752

[CIT0274] Suzuki N, Rivero RM, Shulaev V, Blumwald E, Mittler R 2014 Abiotic and biotic stress combinations. New Phytologist 203, 32–43. 24720847 10.1111/nph.12797

[CIT0275] Tack J, Lingenfelser J, Jagadish SVK 2017 Disaggregating sorghum yield reductions under warming scenarios exposes narrow genetic diversity in US breeding programs. Proceedings of the National Academy of Sciences, USA 114, 9296–9301. 10.1073/pnas.1706383114 PMC5584438 28808013

[CIT0276] Tadesse W, Suleiman S, Tahir I, Sanchez-Garcia M, Jighly A, Hagras A, Thabet SH, Baum M 2019 Heat-tolerant QTLs associated with grain yield and its components in spring bread wheat under heat-stressed environments of Sudan and Egypt. Crop Science 59, 199.

[CIT0277] Takahashi F, Shinozaki K 2019 Long-distance signaling in plant stress response. Current Opinion in Plant Biology 47, 106–111. 30445314 10.1016/j.pbi.2018.10.006

[CIT0278] Talukder SK, Babar MA, Vijayalakshmi K, Poland J, Prasad PV, Bowden R, Fritz A 2014 Mapping QTL for the traits associated with heat tolerance in wheat (*Triticum aestivum* L.). BMC Genetics 15, 97. 25384418 10.1186/s12863-014-0097-4 PMC4234900

[CIT0279] Tang R, Zhu W, Song X, Lin X, Cai J, Wang M, Yang Q 2016 Genome-wide identification and function analyses of heat shock transcription factors in potato. Frontiers in Plant Science 7, 490. 27148315 10.3389/fpls.2016.00490 PMC4836240

[CIT0280] Tao L-X, Tan H-J, Wang X, Cao L-Y, Song J, Cheng S-H 2008 Effects of high-temperature stress on flowering and grain-setting characteristics of guodao 6. Acta Agronomica Sinica 34, 609–674.

[CIT0281] Tayade R, Nguyen T, Oh SA, Hwang YS, Yoon IS, Deshmuk R, Jung K-H, Park SK 2018 Effective strategies for enhancing tolerance to high-temperature stress in rice during the reproductive and ripening stages. Plant Breeding and Biotechnology 6, 1–18.

[CIT0282] Templer SE, Ammon A, Pscheidt D, et al. 2017 Metabolite profiling of barley flag leaves under drought and combined heat and drought stress reveals metabolic QTLs for metabolites associated with antioxidant defense. Journal of Experimental Botany 68, 1697–1713. 28338908 10.1093/jxb/erx038 PMC5441916

[CIT0283] Tenorio FA, Ye C, Redoña E, Sierra S, Laza M, Argayoso MA 2013 Screening rice genetic resources for heat tolerance. SABRAO Journal of Breeding and Genetics 45, 371–381.

[CIT0284] Thomason K, Babar MA, Erickson JE, Mulvaney M, Beecher C, MacDonald G 2018 Comparative physiological and metabolomics analysis of wheat (*Triticum aestivum* L.) following post-anthesis heat stress. PLoS One 13, e0197919. 29897945 10.1371/journal.pone.0197919 PMC5999278

[CIT0285] Thudi M, Upadhyaya HD, Rathore A, et al. 2014 Genetic dissection of drought and heat tolerance in chickpea through genome-wide and candidate gene-based association mapping approaches. PLoS One 9, e96758. 24801366 10.1371/journal.pone.0096758 PMC4011848

[CIT0286] Thussagunpanit J, Jutamanee K, Kaveeta L, Chai-arree W, Pankean P, Homvisasevongsa S, Suksamrarn A 2015 Comparative effects of brassinosteroid and brassinosteroid mimic on improving photosynthesis, lipid peroxidation, and rice seed set under heat stress. Journal of Plant Growth Regulation 34, 320–331.

[CIT0287] Trapero-Mozos A, Morris WL, Ducreux LJM, McLean K, Stephens J, Torrance L, Bryan GJ, Hancock RD, Taylor MA 2018 Engineering heat tolerance in potato by temperature-dependent expression of a specific allele of HEAT-SHOCK COGNATE 70. Plant Biotechnology Journal 16, 197–207. 28509353 10.1111/pbi.12760 PMC5785350

[CIT0288] Uauy C 2017 Wheat genomics comes of age. Current Opinion in Plant Biology 36, 142–148. 28346895 10.1016/j.pbi.2017.01.007

[CIT0289] Valdés-López O, Batek J, Gomez-Hernandez N, et al. 2016 Soybean roots grown under heat stress show global changes in their transcriptional and proteomic profiles. Frontiers in Plant Science 7, 517. 27200004 10.3389/fpls.2016.00517 PMC4843095

[CIT0290] Verdeprado H, Kretzschmar T, Begum H, Raghavan C, Joyce P, Lakshmanan P, Cobb JN, Collard BCY 2018 Association mapping in rice: basic concepts and perspectives for molecular breeding. Plant Production Science 21, 159–176.

[CIT0291] Viana AP, de Resende MDV, Riaz S, Walker MA, Viana AP, de Resende MDV, Riaz S, Walker MA 2016 Genome selection in fruit breeding: application to table grapes. Scientia Agricola 73, 142–149.

[CIT0292] Vierling E 1991 The roles of heat shock proteins in plants. Annual Review of Plant Physiology and Plant Molecular Biology 42, 579–620.

[CIT0293] Vijayalakshmi K, Fritz AK, Paulsen GM, Bai G, Pandravada S, Gill BS 2010 Modeling and mapping QTL for senescence-related traits in winter wheat under high temperature. Molecular Breeding 26, 163–175.

[CIT0294] Vriet C, Hennig L, Laloi C 2015 Stress-induced chromatin changes in plants: of memories, metabolites and crop improvement. Cellular and Molecular Life Sciences 72, 1261–1273. 25578097 10.1007/s00018-014-1792-z PMC11113909

[CIT0295] Vu HS, Tamura P, Galeva NA, Chaturvedi R, Roth MR, Williams TD, Wang X, Shah J, Welti R 2012 Direct infusion mass spectrometry of oxylipin-containing Arabidopsis membrane lipids reveals varied patterns in different stress responses. Plant Physiology 158, 324–339. 22086419 10.1104/pp.111.190280 PMC3252110

[CIT0296] Wahid A, Gelani S, Ashraf M, Foolad M 2007 Heat tolerance in plants: an overview. Environmental and Experimental Botany 61, 199–223.

[CIT0297] Wang J, Gan YT, Clarke F, McDonald CL 2006 Response of chickpea yield to high temperature stress during reproductive development. Crop Science 46, 2171–2178.

[CIT0298] Wang L, Ma H, Song L, Shu Y, Gu W 2012 *a* Comparative proteomics analysis reveals the mechanism of pre-harvest seed deterioration of soybean under high temperature and humidity stress. Journal of Proteomics 75, 2109–2127. 22270011 10.1016/j.jprot.2012.01.007

[CIT0299] Wang L, Shen W, Kazachkov M, Chen G, Chen Q, Carlsson AS, Stymne S, Weselake RJ, Zou J 2012 *b* Metabolic interactions between the Lands cycle and the Kennedy pathway of glycerolipid synthesis in Arabidopsis developing seeds. The Plant Cell 24, 4652–4669. 23150634 10.1105/tpc.112.104604 PMC3531858

[CIT0300] Wang N, Wang Y, Tian F, King GJ, Zhang C, Long Y, Shi L, Meng J 2008 A functional genomics resource for Brassica napus: development of an EMS mutagenized population and discovery of FAE1 point mutations by TILLING. New Phytologist 180, 751–765. 18811617 10.1111/j.1469-8137.2008.02619.x

[CIT0301] Wang T, Uauy C, Till B, Liu CM 2010 TILLING and associated technologies. Journal of Integrative Plant Biology 52, 1027–1030. 20977660 10.1111/j.1744-7909.2010.00999.x

[CIT0302] Wang X, Dinler BS, Vignjevic M, Jacobsen S, Wollenweber B 2015 Physiological and proteome studies of responses to heat stress during grain filling in contrasting wheat cultivars. Plant Science 230, 33–50. 25480006 10.1016/j.plantsci.2014.10.009

[CIT0303] Wang X, Xu Y, Hu Z, Xu C 2018 Genomic selection methods for crop improvement: current status and prospects. The Crop Journal 6, 330–340.

[CIT0304] Wang X, Yan B, Shi M, Zhou W, Zekria D, Wang H, Kai G 2016 Overexpression of a *Brassica campestris* HSP70 in tobacco confers enhanced tolerance to heat stress. Protoplasma 253, 637–645. 26298102 10.1007/s00709-015-0867-5

[CIT0305] Wang Y, Sun F, Cao H, Peng H, Ni Z, Sun Q, Yao Y 2012 TamiR159 directed wheat TaGAMYB cleavage and its involvement in anther development and heat response. PLoS One 7, e48445. 23133634 10.1371/journal.pone.0048445 PMC3486836

[CIT0306] Waters ER 2013 The evolution, function, structure, and expression of the plant sHSPs. Journal of Experimental Botany 64, 391–403. 23255280 10.1093/jxb/ers355

[CIT0307] Waters ER, Vierling E 1999 Chloroplast small heat shock proteins: evidence for atypical evolution of an organelle-localized protein. Proceedings of the National Academy of Sciences, USA 96, 14394–14399. 10.1073/pnas.96.25.14394 PMC24447 10588716

[CIT0308] Weichert H, Högy P, Mora-Ramirez I, Fuchs J, Eggert K, Koehler P, Weschke W, Fangmeier A, Weber H 2017 Grain yield and quality responses of wheat expressing a barley sucrose transporter to combined climate change factors. Journal of Experimental Botany 68, 5511–5525. 29069444 10.1093/jxb/erx366 PMC5853912

[CIT0309] Welti R, Shah J, Li W, Li M, Chen J, Burke JJ, Fauconnier ML, Chapman K, Chye ML, Wang X 2007 Plant lipidomics: discerning biological function by profiling plant complex lipids using mass spectrometry. Frontiers in Bioscience 12, 2494–2506. 17127258 10.2741/2250

[CIT0310] Wen J, Jiang F, Weng Y, Sun M, Shi X, Zhou Y, Yu L, Wu Z 2019 Identification of heat-tolerance QTLs and high-temperature stress-responsive genes through conventional QTL mapping, QTL-seq and RNA-seq in tomato. BMC Plant Biology 19, 398. 31510927 10.1186/s12870-019-2008-3 PMC6739936

[CIT0311] Wiese S, Reidegeld KA, Meyer HE, Warscheid B 2007 Protein labeling by iTRAQ: a new tool for quantitative mass spectrometry in proteome research. Proteomics 7, 340–350. 17177251 10.1002/pmic.200600422

[CIT0312] Wu X, Gong F, Cao D, Hu X, Wang W 2016 Advances in crop proteomics: PTMs of proteins under abiotic stress. Proteomics 16, 847–865. 26616472 10.1002/pmic.201500301

[CIT0313] Wu X, Gong F, Yang L, Hu X, Tai F, Wang W 2014 Proteomic analysis reveals differential accumulation of small heat shock proteins and late embryogenesis abundant proteins between ABA-deficient mutant vp5 seeds and wild-type Vp5 seeds in maize. Frontiers in Plant Science 5, 801. 25653661 10.3389/fpls.2014.00801 PMC4299431

[CIT0314] Xu C, Fan J, Cornish AJ, Benning C 2008 Lipid trafficking between the endoplasmic reticulum and the plastid in Arabidopsis requires the extraplastidic TGD4 protein. The Plant Cell 20, 2190–2204. 18689504 10.1105/tpc.108.061176 PMC2553622

[CIT0315] Xu J, Driedonks N, Rutten MJM, Vriezen WH, de Boer GJ, Rieu I 2017 Mapping quantitative trait loci for heat tolerance of reproductive traits in tomato (*Solanum lycopersicum*). Molecular Breeding 37, 58. 28479863 10.1007/s11032-017-0664-2 PMC5395597

[CIT0316] Xu W, Cai SY, Zhang Y, et al. 2016 Melatonin enhances thermotolerance by promoting cellular protein protection in tomato plants. Journal of Pineal Research 61, 457–469. 27484733 10.1111/jpi.12359

[CIT0317] Xue GP, Drenth J, McIntyre CL 2015 TaHsfA6f is a transcriptional activator that regulates a suite of heat stress protection genes in wheat (*Triticum aestivum* L.) including previously unknown Hsf targets. Journal of Experimental Botany 66, 1025–1039. 25428996 10.1093/jxb/eru462 PMC4321556

[CIT0318] Xue GP, Sadat S, Drenth J, McIntyre CL 2014 The heat shock factor family from *Triticum aestivum* in response to heat and other major abiotic stresses and their role in regulation of heat shock protein genes. Journal of Experimental Botany 65, 539–557. 24323502 10.1093/jxb/ert399 PMC3904712

[CIT0319] Yamakawa H, Hakata M 2010 Atlas of rice grain filling-related metabolism under high temperature: joint analysis of metabolome and transcriptome demonstrated inhibition of starch accumulation and induction of amino acid accumulation. Plant & Cell Physiology 51, 795–809. 20304786 10.1093/pcp/pcq034 PMC2871029

[CIT0320] Yan J, Yu L, Xuan J, Lu Y, Lu S, Zhu W 2016 De novo transcriptome sequencing and gene expression profiling of spinach (*Spinacia oleracea* L.) leaves under heat stress. Scientific Reports 6, 19473. 26857466 10.1038/srep19473 PMC4746569

[CIT0321] Yang H, Gu X, Ding M, Lu W, Lu D 2018 Heat stress during grain filling affects activities of enzymes involved in grain protein and starch synthesis in waxy maize. Scientific Reports 8, 15665. 30353095 10.1038/s41598-018-33644-z PMC6199321

[CIT0322] Yang X, Zhu W, Zhang H, Liu N, Tian S 2016 Heat shock factors in tomatoes: genome-wide identification, phylogenetic analysis and expression profiling under development and heat stress. PeerJ 4, e1961. 27190703 10.7717/peerj.1961 PMC4867723

[CIT0323] Ye C, Argayoso MA, Redoña ED, et al. 2012 Mapping QTL for heat tolerance at flowering stage in rice using SNP markers. Plant Breeding 131, 33–41.

[CIT0324] Ye C, Tenorio FA, Argayoso MA, Laza MA, Koh HJ, Redoña ED, Jagadish KS, Gregorio GB 2015 Identifying and confirming quantitative trait loci associated with heat tolerance at flowering stage in different rice populations. BMC Genetics 16, 41. 25895682 10.1186/s12863-015-0199-7 PMC4415243

[CIT0325] Yona N 2015 Genetic characterization of heat tolerant (HT) upland mutant rice (Oryza sativa L.) lines selected from rice genotypes. Master thesis, University of Agriculture Morogoro, Tanzania.

[CIT0326] Yousuf PY, Abd_Allah EF, Nauman M, Asif A, Hashem A, Alqarawi AA, Ahmad A 2017 Responsive proteins in wheat cultivars with contrasting nitrogen efficiencies under the combined stress of high temperature and low nitrogen. Genes 8, 356. 10.3390/genes8120356 PMC5748674 29186028

[CIT0327] Yu E, Fan C, Yang Q, Li X, Wan B, Dong Y, Wang X, Zhou Y 2014 Identification of heat responsive genes in *Brassica napus* siliques at the seed-filling stage through transcriptional profiling. PLoS One 9, e101914. 25013950 10.1371/journal.pone.0101914 PMC4094393

[CIT0328] Zaidi SS, Mukhtar MS, Mansoor S 2018 Genome editing: targeting susceptibility genes for plant disease resistance. Trends in Biotechnology 36, 898–906. 29752192 10.1016/j.tibtech.2018.04.005

[CIT0329] Zampieri M, Ceglar A, Dentener F, Toreti A 2017 Wheat yield loss attributable to heat waves, drought and water excess at the global, national and subnational scales. Environmental Research Letters 12, 064008.

[CIT0330] Zang X, Geng X, Wang F, et al. 2017 Overexpression of wheat ferritin gene TaFER-5B enhances tolerance to heat stress and other abiotic stresses associated with the ROS scavenging. BMC Plant Biology 17, 14. 28088182 10.1186/s12870-016-0958-2 PMC5237568

[CIT0331] Zandalinas SI, Balfagón D, Arbona V, Gómez-Cadenas A 2017 Modulation of antioxidant defense system is associated with combined drought and heat stress tolerance in citrus. Frontiers in Plant Science 8, 953. 28638395 10.3389/fpls.2017.00953 PMC5461256

[CIT0332] Zhang B, VerBerkmoes NC, Langston MA, Uberbacher E, Hettich RL, Samatova NF 2006 Detecting differential and correlated protein expression in label-free shotgun proteomics. Journal of Proteome Research 5, 2909–2918. 17081042 10.1021/pr0600273

[CIT0333] Zhang C, Hsieh T-F 2013 Heritable epigenetic variation and its potential applications for crop improvement. Plant Breeding and Biotechnology 1, 307–319.

[CIT0334] Zhang L, Geng X, Zhang H, et al. 2017 Isolation and characterization of heat-responsive gene TaGASR1 from wheat (*Triticum aestivum* L.). Journal of Plant Biology 60, 57–65.

[CIT0335] Zhang L, Kato Y, Otters S, Vothknecht UC, Sakamoto W 2012 Essential role of VIPP1 in chloroplast envelope maintenance in Arabidopsis. The Plant Cell 24, 3695–3707. 23001039 10.1105/tpc.112.103606 PMC3480296

[CIT0335a] Zhao Q, Chen W, Bian J, et al. 2018 Proteomics and phosphoproteomics of heat stress-responsive mechanisms in Spinach. Frontiers in Plant Science 9. 10.3389/fpls.2018.00800 PMC6029058 29997633

[CIT0336] Zhao C, Liu B, Piao S, et al. 2017 Temperature increase reduces global yields of major crops in four independent estimates. Proceedings of the National Academy of Sciences, USA 114, 9326–9331. 10.1073/pnas.1701762114 PMC5584412 28811375

[CIT0337] Zhao F, Zhang D, Zhao Y, Wang W, Yang H, Tai F, Li C, Hu X 2016 The difference of physiological and proteomic changes in maize leaves adaptation to drought, heat, and combined both stresses. Frontiers in Plant Science 7, 1471. 27833614 10.3389/fpls.2016.01471 PMC5080359

[CIT0338] Zhang X, Högy P, Wu X, Schmid I, Wang X, Schulze WX, Jiang D, Fangmeier A 2018 Physiological and proteomic evidence for the interactive effects of post-anthesis heat stress and elevated CO_2_ on wheat. Proteomics 18, 1800262. 10.1002/pmic.201800262 30307109

[CIT0339] Zheng G, Tian B, Zhang F, Tao F, Li W 2011 Plant adaptation to frequent alterations between high and low temperatures: remodelling of membrane lipids and maintenance of unsaturation levels. Plant, Cell & Environment 34, 1431–1442. 10.1111/j.1365-3040.2011.02341.x PMC3980542 21486310

[CIT0340] Zou J, Liu C, Chen X 2011 Proteomics of rice in response to heat stress and advances in genetic engineering for heat tolerance in rice. Plant Cell Reports 30, 2155–2165. 21769604 10.1007/s00299-011-1122-y

